# The Brain Anatomy of the Brown Bear (Carnivora, *Ursus arctos* L., 1758) Compared to That of Other Carnivorans: A Cross-Sectional Study Using MRI

**DOI:** 10.3389/fnana.2019.00079

**Published:** 2019-08-29

**Authors:** Tomasz Sienkiewicz, Agnieszka Sergiel, Djuro Huber, Robert Maślak, Marcin Wrzosek, Przemysław Podgórski, Slaven Reljić, Łukasz Paśko

**Affiliations:** ^1^Department of Evolutionary Biology and Conservation of Vertebrates, Institute of Environmental Biology, Faculty of Biological Sciences, University of Wrocław, Wrocław, Poland; ^2^Department of Wildlife Conservation, Institute of Nature Conservation, Polish Academy of Sciences, Krakow, Poland; ^3^Department of Biology, Faculty of Veterinary Medicine, University of Zagreb, Zagreb, Croatia; ^4^Department of Internal Medicine and Clinic of Diseases for Horses, Dogs and Cats, Faculty of Veterinary Medicine, Wrocław University of Environmental and Life Sciences, Wrocław, Poland; ^5^Department of General Radiology, Interventional Radiology and Neuroradiology, Faculty of Postgraduate Medical Training, Wrocław Medical University, Wrocław, Poland

**Keywords:** comparative neuroanatomy, ursids, brain imaging, brown bear (*Ursus arctos*), Carnivora

## Abstract

In this study, we aimed to provide a neuroanatomy atlas derived from cross-sectional and magnetic resonance imaging (MRI) of the encephalon of the brown bear (*Ursus arctos*). A postmortem brain analysis using magnetic resonance imaging (MRI – 1,5T; a high-resolution submillimeter three-dimensional T1-3D FFE) and cross-sectional macroscopic anatomy methods revealed major embryological and anatomical subdivisions of the encephalon, including the ventricular system. Most of the internal structures were comparably identifiable in both methods. The tractus olfactorius medialis, corpus subthalamicum, brachium colliculi rostralis, fasciculus longitudinalis medialis, nuclei vestibulares, velum medullare rostrale, nucleus fastigii, fasciculi cuneatus et gracilis were identified entirely by cross-sectional macroscopic analysis. However, the glandula pinealis, lemniscus lateralis and nuclei rhaphe were visualized only with MRI. Gross neuroanatomic analysis provided information about sulci and gyri of the cerebral hemispheres, components of the vermis and cerebellar hemispheres, and relative size and morphology of constituents of the rhinencephalon and cerebellum constituents. Similarities and discrepancies in identification of structures provided by both methods, as well as hallmarks of the structures facilitating identification using these methods are discussed. Finally, we compare the brown bear encephalon with other carnivores and discuss most of the identified structures compared to those of the domestic dog, the domestic cat, Ursidae and Mustelidae families and Pinnipedia clade.

## Introduction

Relatively little is known about Ursidae brains’ anatomy, in comparison with common domestic animals – the dog (*Canis lupus familiaris*), the cat (*Felis catus*) and the ferret (*Mustela putorius furo*). Among Carnivora, the domestic cat and domestic dog have been the most favored for an extensive neuroanatomical research employing the newest imaging techniques (e.g., Leigh et al., 2008; Mogicato et al., 2011b; Gray-Edwards et al., 2014), while for numerous species merely brain surface anatomy is known.

Most of the neuroanatomical analyses conducted so far have focused on sulci and gyri patterns in various Carnivora families (e.g., Mettler and Goss, 1946; Radinsky, 1973b, a, 1975). Some studies can provide detailed information about internal brain structures due to employment of magnetic resonance imaging (MRI) combined with cross-sectional analyses. MRI method has been in use for about 20 years in studies of central nervous system features of given Carnivora species. An internal anatomical description of the canine brain using MRI (with 1,5 tesla magnet – 1,5T) was performed by Leigh et al. (2008). There are also studies focused on comparison of the amount of data acquired with employment of different magnetic field MRI scanners: 1.5T to 7T (e.g., Kang et al., 2009) and 3T to 7T (e.g., Martín-Vaquero et al., 2010). Mogicato et al. (2011b) compared three transections using three methods: unstained and stained cross-sections, and virtual sections acquired by MRI (1T). Also the cranial nerves nearest to the brain parts with cranial foramina were investigated using MRI method (Couturier et al., 2005) Additionally, MRI techniques have been used to create neuroanatomical atlases for certain species. An innovative atlas of digitally smoothed canine brain surface showing area underneath the brain sulci using MRI has been performed by Datta et al. (2012), while online brain atlas published by Minnesota College of Veterinary Medicine (Fletcher, 2007) contains stained brain cross-sections and its MRI-acquired counterparts. Out of wild canids, an MRI and cross-sectional brain atlas of the red fox (*Vulpes vulpes*) has been created (Kassab and Bahgat, 2007). Sawada et al. (2013) studied the sexual dimorphism of neuroanatomical traits of the ferret telencephalon with use of MRI (7T) and immunohistochemistry, leading to a conclusion that total brain volume, cerebral cortex volume and subcortical white matter were bigger in males. The authors described the cerebral cortex’s cytoarchitectonics by delineating motor and sensory areas of the pallium dorsale and the layers of bulbus olfactorius and visualized it in the colored map created with MRI- and immunohistochemistry-acquired brain slides. Neal et al. (2007) used MRI to trace gyrification in the ferret with 4.7T and 8.5T magnetic strength. They visualized postnatal gradation of the sulci revealed toward the brain midline. Furthermore, postnatal neuronal proliferation, migration and level of myelination were studied on the basis of signal differences at T2 in the selected cortex and periventricular areas. It was shown that the growth rate of cortical volume exceeds the growth rate of the other analyzed subcortical structures. Barnette et al. (2009) studied alteration in MRI (4.7T) signal intensity during different stages of gyrification and reconstructed a 3D map of the gyri curvatures followed by comparison of gyrification in the ferret and human fetus. A featured brain MRI atlas (T2, 1,5T) of the California sea lion (*Zalophus californianus*) containing 20 transverse and 14 oblique cross-sectional MRI images was published by Montie et al. (2009). Many crucial brain components were depicted, and Pinnipedia signature traits were corroborated. MRI brain anatomy atlases were also developed for the cat (Yamada et al., 1994; Mogicato et al., 2011b; Gray-Edwards et al., 2014), and additionally combined with computer tomography (CT) to reveal the anatomical relation of cranial nerves with the brain and skull foramina (Gomes et al., 2009). A general brain anatomy of the young Bengal tiger (*Panthera tigris tigris*) could be also revealed by MRI (1.5T) in T1, T2 and diffusion MRI (DWI), and showed its utility for clinical assessment in veterinary neurology (Snow et al., 2004). As emphasized by this paragraph, MRI allowed revealing general and species-specific neuroanatomical traits among carnivores, supplementing knowledge acquired with traditional cross sections, while for some species the internal brain anatomy remains virtually unknown. Neuroanatomical features and volumetric analysis possible with MRI techniques available for all carnivores species, would allow inter- and intra-specific comparisons and its translation into functions in behavior and ecology of Carnivora.

Bears have evolved in Carnivora clade (Flynn et al., 2005; Supplementary Figure S1) during the late Oligocene and early Miocene, about 20–25 million years before present (McLellan and Reiner, 1994), attaining a wide geographical distribution range (Kumar et al., 2017). Family Ursidae comprises eight extant species, classified into three subfamilies: Ursinae (with polar bear *Ursus maritimus*, brown bear *Ursus arctos*, American black bear *Ursus americanus*, Asiatic black bear *Ursus thibetanus*, sloth bear *Melursus ursinus*, and Malayan sun bear *Helarctos malayanus*), Ailuropodinae (with giant panda *Ailuropoda melanoleuca*) and Tremarctinae (with Andean bear *Tremarctos ornatus*) (Wagner, 2010). Morphologically and taxonomically, bears possess all the traits of carnivores but, with the exception of the polar bear, have diets often comprised primarily of plant matter. A generalist omnivore strategy most of the bear species evolved with, allows them to successfully occupy a broad array of habitats (Robbins et al., 2004). Diverse habitats and its physical characteristics create challenges that influenced evolution of brain and sensory structures in bears as they have to detect through complex environment (e.g., Togunov et al., 2017). Additionally, bears are solitary and non-territorial (Bellemain et al., 2006) and as such, they benefit from conveying multimodal cues to expedite breeding season, to recognize family and kin, and to avoid conspecifics. Available anatomical, histological and behavioral evidence suggests that bears are somatosensory specialists (Bacon and Burghardt, 1974; Nachtigall et al., 2007; Pastor et al., 2008; Owen and Bowles, 2011; Togunov et al., 2017; Tomiyasu et al., 2017), and possess neuroanatomical correlates for significant sensitivity of their extremities (Kamiya and Pirlot, 1988b).

We have performed analysis of a detailed brain atlas of the brown bear, from countable internal anatomy neurostructures acquired by two methods – macroscopic anatomy and MRI. We produced a series of virtual slices from MRI of postmortem brain, in which detailed structures could be recognized and then compared to them in manually performed cross-sections. Numerous structures have been recognized using both methods, and allow comparing to what extent the MRI scanning can reflect actual shape and expanse of given structure observed in cross sections. The atlas may be useful to veterinarians, as it is one of very few extensive sources of knowledge about the brain of a wild Carnivora species. In this paper we also present a comparative analysis of *U. arctos* brain with other Carnivora species, as well as with specimens available in Comparative Mammalian Brain Collections to further contribute to our understanding of brain organization. Those observations might be also used to infer ecological aspects that stem from particular brain traits.

## Materials and Methods

### Animals and Brain Tissue Preparation

For the present study, brains were acquired from traffic-killed specimens collected within a frame of Brown Bear Management Plan for the Republic of Croatia (Huber et al., 2008). The use of tissues from animals found dead is exempt from approval by Ethical Committee for Animal Experimentation. The laboratory procedures began from brain immersion-fixing in 4% buffered formaldehyde immediately after its removal from the skull. Table 1 contains sex, age, and collection site data for each specimen. The age was determined using premolar teeth cementum age determination method (Matson et al., 1993). The brain of the bear RH 178-15 was used as a model brain for the analyses due to its best quality in terms of tissue preservation.

**TABLE 1:** Data on specimens used in the study (Supplementary Figures S2–S4).

**Specimen ID**	**Sex**	**Determined age [years]**	**Collection site**	**Date and time of fixation**
RH0178-15	♀	2	Railway track, Tièevo – Gomirje; Dobra VIII/28	13.09.2015; 05:05 p.m.
RH0156/13	♀	3	Railway track, tunel Bukovac, Bjelolasica VIII/2	25.06.2013; 08:00 a.m.
RH155/14	♂	3	Road, Plitvice Lakes National Park	14.05.2014; 04:30 p.m.
RH194/14	♀	1	Railway track, Požari – Meðuvoðe, E IV/123	5.10.2014; 09:00 a.m.

*Brain of the specimen RH0178-15 was used as a model brain for MRI imaging and cross-sections.*

### Magnetic Resonance Imaging

The isolated brain of RH0178-15 was imaged at Centre for Experimental Diagnostics and Biomedical Innovations in Wrocław. MRI scanning of the brain was carried out on a 1.5-T Philips MRI scanner. 3D Fast Field Echo (FFE) T1-weighted images in the transverse planes were acquired in isotropic resolution with the following parameters: slice thickness 0,33 mm; matrix 660 × 660; FOV (field-of-view) 22 cm; TE (echo time) 9,7; TR (repetition time) 25,0; Flip Angle 30. After scanning, the brain was re-immersed in 4% buffered formaldehyde and sectioned within 24 h. Visualization of MRI images was performed with Medical Imaging Interaction Toolkit (MITK) software and images in different planes were selected to make the best correspondence with anatomical cross-sections. MRI. The recognition of examined structures was performed on the basis of previously examined anatomical cross-sections and also using MRI brain atlas of the dog (Leigh et al., 2008) and *Zalophus californianus* (Montie et al., 2009). Photo processing was performed with Canvas X 2017 GIS software with data acquired directly from MITK software.

### Macroscopic Anatomy

Before examination, brains had been irrigated with running water for 24–48 h. The measurements of RH0178-15 specimen were performed with a caliper (Table 2). Subsequently, the RH0178-15 brain was cross-sectioned into 5-mm-thick sections with a stainless-steel knife. The examination was performed with use of Nikon SMZ800 stereomicroscope, overhead magnifying glass (3,5x) and with the naked eye. The images were taken with reflex camera Nikon D80. An internal preservation of the remaining specimens was of lower quality than the brain RH0178-15. Therefore, they were used as the point of reference for the examination of the brain surface variability and gyrification pattern and some other dubious traits mentioned in the text, including presence of the septum pellucidum, and were not analyzed in all details. The photo processing was performed with Canvas X 2017 GIS software. Structures of interest were identified by comparison with available atlases of the dog brain (Meyer, 1964; Whalen, 2003; Fletcher, 2007; Leigh et al., 2008; Uemura, 2015), textbooks (Meyer, 1964; Kobryń and Kobryńczuk, 2004; Uemura, 2015), and previously published observations of bear brains (Mettler and Goss, 1946; Kamiya and Pirlot, 1988b). Comparisons with other Carnivora were performed with illustrations in above mentioned texts and specimens available in Comparative Mammalian Brain Collections (CMBC).

**TABLE 2:** Gross measurements of studied specimens.

**Measurement**	**Value [mm]**			
	**RH0178-15**	**RH0156/13**	**RH155/14**	**RH194/14**
Encephalon – maximum length^1^	119	111	116	102
Encephalon – maximum width^2^	86	96	85	95
Encephalon – maximum height^2^	55	56	102	85
Telencephalon – maximum length^3^	103	97	102	89
Right cerebral hemisphere – maximum length	98	93	104	87
Left cerebral hemisphere – maximum length	102	98	70	64
Cerebellum maximum width	66	63	50	48

*^1^The measurement at the basis of the encephalon from the most caudal to the most rostral edge. ^2^The measurement at the level of the temporal lobes. ^3^The measurement at the level of the fissura longitudinalis.*

### Nomenclature

Most of nomenclature was acquired from Nomina Anatomica Veterinaria 5th edition (International Committee on Veterinary Gross Anatomical Nomenclature [ICVGAN], 2005). The remainder came from cited literature (Mettler and Goss, 1946; Meyer, 1964). The source of each term is shown in Table 3. Pallium nomenclature comes from Comparative Vertebrate Neuroanatomy – Evolution and Adaptation (Butler and Hodos, 2005). For pallium mediale homologs we used term “hippocampal formation” to remove a common ambiguity concerning the term “hippocampus”; using *sensu lato* (all cortical structures) or *sensu stricto* (cornu ammonis). “Hippocampal formation” stands for the subiculum, cornu ammonis and gyrus dentatus with associated white matter – fimbriae and alveus hippocampi. “Rhinencephalon” in our study stands for primary olfactory structures (bulbus olfactorius, pedunculus olfactorius, tractus olfactorius) and associated superficial pallium laterale structures (olfactory cortex, lobus piriformis, gyrus olfactorius lateralis, tuberculum olfactorium). The English and Latin names of species mentioned in the paper come from Cichocki et al. (2015).

**TABLE 3:** Nomenclature used in the study.

**Abbreviation**	**Latin term**	**English term**	**Figures**	**References**
AH	Alveus hippocampi	Alveus of hippocampus	6, 7, 8	NAV^5th^; Welento, 2002
AqC	Aquaeductus cerebri	Cerebral aqueduct	7, 8, 9	NAV^5th^; Welento, 2002
ATh	Adhaesio interthalamica	Interthalamic adhesion	6, 7	NAV^5th^; Welento, 2002
BCoC	Brachium colliculi caudalis	Brachium of caudal colliculus	8, 9	NAV^5th^; Welento, 2002
BCoR	Brachium colliculi rostralis	Brachium of rostral colliculus	8	NAV^5th^; Welento, 2002
BO	Bulbus olfactorius	Olfactory bulb	1, 2	NAV^5th^; Welento, 2002
BV	Vasa sanguinea	Blood vessel	1, 4, 5	NAV^5th^; Welento, 2002
CA	Cornu ammonis	Ammon’s horn	6, 7, 8	NAV^5th^; Welento, 2002
CAg	Corpus amygdaloideum	Amygdaloid body	6	NAV^5th^; Welento, 2002
CC	Corpus callosum	Corpus callosum	4, 5, 6, 7, 8	NAV^5th^; Welento, 2002
CCa	Commissura caudalis	Caudal commissure	7	NAV^5th^; Welento, 2002
CCoC	Commissura colliculorum caudalium	Commissure of caudal colliculi	9	NAV^5th^; Welento, 2002
CCoR	Commissura colliculorum rostralium	Commissure of rostral colliculi	8	NAV^5th^; Welento, 2002
CE	Capsula externa	External capsule	4, 5, 7	NAV^5th^; Welento, 2002
CEx	Capsula extrema	Extreme capsule	4, 5, 7	NAV^5th^; Welento, 2002
CFx	Corpus fornicis	Body of fornix	6	NAV^5th^; Welento, 2002
CGL	Corpus geniculatum laterale	Lateral geniculate body	7, 8	NAV^5th^; Welento, 2002
CGM	Corpus geniculatum mediale	Medial geniculate body	7, 8	NAV^5th^; Welento, 2002
CI	Capsula interna	Internal capsule	4, 5, 6, 7	NAV^5th^; Welento, 2002
ClFx	Columna fornicis	Column of fornix	6	NAV^5th^; Welento, 2002
Cm	Claustrum	Claustrum	4, 5, 6, 7	NAV^5th^; Welento, 2002
CMa	Corpus mamillare	Mamillary body	1, 7	NAV^5th^; Welento, 2002
CnD	Cornu dorsale	Dorsal horn	12	NAV^5th^; Welento, 2002
CnV	Cornu ventrale	Ventral horn	12	NAV^5th^; Welento, 2002
CoC	Colliculus caudalis	Caudal colliculus	8, 9	NAV^5th^; Welento, 2002
COp	Chiasma opticum	Optic chiasm	1, 5, 6	NAV^5th^; Welento, 2002
CoR	Colliculus rostralis	Rostral colliculus	8, 9	NAV^5th^; Welento, 2002
CrC	Crus cerebri	Cerebral crus	7, 8, 9	NAV^5th^; Welento, 2002
CrFx	Crus fornicis	Crus of fornix	6, 7	NAV^5th^; Welento, 2002
CSu	Corpus subthalamicum	Subthalamic body	7	NAV^5th^; Welento, 2002
CT	Corpus trapezoideum	Trapezoid body	10	NAV^5th^; Welento, 2002
Cu	Culmen	Culmen	1, 9, 10, 11	NAV^5th^; Welento, 2002
CxC	Cortex cerebelli	Cerebellar cortex	9, 10, 11, 12	NAV^5th^; Welento, 2002
Dc	Declive	Declive	1, 11	NAV^5th^; Welento, 2002
DLM	Decussatio lemniscorum medialium	Decussation of medial lemniscus	11	NAV^5th^; Welento, 2002
DPCR	Decussatio pedunculorum cerebellarium rostralium	Decussation of rostral cerebellar peduncles	8, 9	NAV^5th^; Welento, 2002
DPy	Decussatio pyramidum	Decussation of pyramids	12	NAV^5th^; Welento, 2002
FaC	Fasciculus cuneatus	Cuneate fasciculus	12	NAV^5th^; Welento, 2002
FaG	Fasciculus gracilis	Gracile fasciculus	12	NAV^5th^; Welento, 2002
Fc	Flocculus	Flocculus	10	NAV^5th^; Welento, 2002
FIp	Fossa interpeduncularis	Interpeduncular fossa	1, 8	NAV^5th^; Welento, 2002
FIv	Foramen interventriculare	Interventricular foramen	6	NAV^5th^; Welento, 2002
FH	Fimbria hippocampi	Fimbria of hippocampus	7	NAV^5th^; Welento, 2002
FLC	Fissura longitudinalis cerebri	Longitudinal cerebral fissura	1, 2, 3, 4, 5, 6, 7, 8, 9	NAV^5th^; Welento, 2002
FLM	Fasciculus longitudinalis medialis	Medial longitudinal fasciculus	8, 9	NAV^5th^; Welento, 2002
FP	Fissura pseudosylvia	Pseudosylvia fissure	1, 5, 6	NAV^5th^; Welento, 2002
FPoT	Fibrae pontis transversae	Transverse pontine fibers	8, 9	NAV^5th^; Welento, 2002
FPr	Fissura prima	Primary fissure	1, 9, 10, 11	NAV^5th^; Welento, 2002
FRM	Formatio reticularis mesencephali	Reticular formation of midbrain	8, 9	NAV^5th^; Welento, 2002
FRMO	Formatio reticularis medulla oblongatae	Reticular formation of medulla oblongata	10, 11	NAV^5th^; Welento, 2002
FRPo	Formatio reticularis pontis	Reticular formation of pons	9	NAV^5th^; Welento, 2002
FuD	Funiculus dorsalis	Dorsal funiculus	12	NAV^5th^; Welento, 2002
FuL	Funiculus lateralis	Lateral funiculus	12	NAV^5th^; Welento, 2002
FuV	Funiculus ventralis	Ventral funiculus	12	NAV^5th^; Welento, 2002
FVm	Folium vermis	Folium of vermis	1, 12	NAV^5th^; Welento, 2002
Fx	Fornix	Fornix	6, 7	NAV^5th^; Welento, 2002
GCC	Gyrus compositus caudalis	Caudal composite gyrus	1	NAV^5th^; Welento, 2002
GCi	Gyrus cinguli	Cingulate gyrus	4, 5, 6, 7, 8	NAV^5th^; Welento, 2002
GD	Gyrus dentatus	Dentate gyrus	7, 8	NAV^5th^; Welento, 2002
GEm	Gyrus ectomarginalis	Ectomarginal gyrus	1, 8, 9	NAV^5th^; Welento, 2002
GEsC	Gyrus ectosylvius caudalis	Caudal ectosylvian gyrus	1, 6, 7, 8	NAV^5th^; Welento, 2002
GEsR	Gyrus ectosylvius rostralis	Rostral ectosylvian gyrus	1, 4, 5, 6, 7	NAV^5th^; Welento, 2002
GFD	Gyrus frontalis dorsalis	Dorsal frontal gyrus	1, 3, 4, 5, 6	Mettler and Goss, 1946
GFM	Gyrus frontalis medius	Middle frontal gyrus	1, 2, 3, 4	Mettler and Goss, 1946
GFV	Gyrus frontalis ventralis	Ventral frontal gyrus	1, 2, 3	Mettler and Goss, 1946
GlPn	Glandula pinealis	Pineal gland	7	NAV^5th^; Welento, 2002
GM	Gyrus marginalis	Marginal gyrus	1, 6, 7, 8	NAV^5th^; Welento, 2002
GOc	Gyrus occipitalis	Occipital gyrus	1	NAV^5th^; Welento, 2002
GOL	Gyrus olfactorius lateralis	Lateral olfactory gyrus	1, 3, 4, 5	NAV^5th^; Welento, 2002
GP	Globus pallidus	Globus pallidus	4, 5, 6	NAV^5th^; Welento, 2002
GPc	Gyrus postcruciatus	Postcruciate gyrus	1, 5, 6	NAV^5th^; Welento, 2002
GPh	Gyrus parahippocampalis	Parahippocampal gyrus	8	NAV^5th^; Welento, 2002
GPr	Gyrus proreus	Prorean gyrus	1, 2	NAV^5th^; Welento, 2002
GS	Gyrus splenialis	Splenial gyrus	7, 8, 9	Meyer, 1964
GSC	Gyrus sigmoideus caudalis	Caudal sigmoid gyrus	1, 4, 5, 6	Meyer, 1964
GSR	Gyrus sigmoideus rostralis	Rostral sigmoid gyrus	1, 2, 3, 4	Meyer, 1964
GSsC	Gyrus suprasylvius caudalis	Caudal suprasylvian gyrus	1, 7, 8	Meyer, 1964
GSsR	Gyrus suprasylvius rostralis = gyrus coronalis	Rostral suprasylvian gyrus = coronal gyrus	1, 3, 4, 5, 6, 7, 8	Meyer, 1964
Ha	Habenula	Habenula	7	NAV^5th^; Welento, 2002
HC	Hemisphaerium cerebelli	Cerebellar hemispheres	1, 9, 10, 11, 12	NAV^5th^; Welento, 2002
HD	Hippocampus dorsalis	Dorsal hippocampus	7, 8	Butler and Hodos, 2005
HF	*b*	Hippocampal formation	6, 7, 8	Butler and Hodos, 2005
NHOv	Hilus nuclei olivaris	Hilum of olivary nucleus	10, 11	NAV^5th^; Welento, 2002
If	Infundibulum	Infundibulum	1	NAV^5th^; Welento, 2002
LA	Lobulus ansiformis	Ansiform lobule	1, 9, 10, 11, 12	NAV^5th^; Welento, 2002
LACC	Lobulus ansiformis – crus caudale	Ansiform lobule – caudal crus	1, 11, 12	NAV^5th^; Welento, 2002
LACR	Lobulus ansiformis – crus rostrale	Ansiform lobule – rostral crus	1, 9, 10, 11, 12	NAV^5th^; Welento, 2002
LiC	Lingula cerebelli	Lingula	10	NAV^5th^; Welento, 2002
LL	Lemniscus lateralis	Lateral lemniscus	9	NAV^5th^; Welento, 2002
LM	Lemniscus medialis	Medial lemniscus	10, 11	NAV^5th^; Welento, 2002
Ln	*b*	Lesion	9	
LP	Lobus piriformis	Piriform lobe	1, 5, 6, 7	NAV^5th^; Welento, 2002
LPm	Lobulus paramedianus	Paramedian lobule	1	NAV^5th^; Welento, 2002
MO	Medulla oblongata	Medulla oblongata	1, 9, 10, 11, 12	NAV^5th^; Welento, 2002
NAc	Nucleus accumbens	Accumbens nucleus	5	NAV^5th^; Welento, 2002
NC	Nucleus caudatus	Caudate nucleus	4, 5, 6, 7, 8	NAV^5th^; Welento, 2002
NCL	Nucleus cuneatus lateralis	Lateral cuneate nucleus	10, 11	NAV^5th^; Welento, 2002
NCM	Nucleus cuneatus medialis	Medial cuneate nucleus	11, 12	NAV^5th^; Welento, 2002
NCoC	Nucleus colliculi caudalis	Nucleus of caudal colliculus	9	NAV^5th^; Welento, 2002
Nd	Nodulus	Nodule	11	NAV^5th^; Welento, 2002
NEp	Nucleus endopeduncularis	Endopeduncular nucleus	6, 7	NAV^5th^; Welento, 2002
NF	Nucleus fastigii	Fastigial nucleus	11	NAV^5th^; Welento, 2002
NG	Nucleus gracilis	Gracile nucleus	11, 12	NAV^5th^; Welento, 2002
NGL	Nucleus geniculatus lateralis	Lateral geniculate nucleus	7, 8	NAV^5th^; Welento, 2002
NGM	Nucleus geniculatus medialis	Medial geniculate nucleus	7, 8	NAV^5th^; Welento, 2002
NH	Nuclei hypothalamici	Hypothalamici nuclei	6	NAV^5th^; Welento, 2002
NIC	Nucleus interpositus cerebelli	Intercalated cerebellar nucleus	10, 11	NAV^5th^; Welento, 2002
NIp	Nucleus interpeduncularis	Interpeduncular nucleus	8	NAV^5th^; Welento, 2002
NLC	Nucleus lateralis cerebelli = nucleus dentatus	Nucleus lateralis cerebelli = dentate nucleus	10, 11	NAV^5th^; Welento, 2002
NMNO	Nucleus motorius n. oculomotorii	Motor nucleus of oculomotor nerve	7, 8	NAV^5th^; Welento, 2002
NOv	Nucleus olivaris	Olivary nucleus	10, 11	NAV^5th^; Welento, 2002
NPo	Nuclei pontis	Pontine nuclei	8, 9	NAV^5th^; Welento, 2002
NR	Nucleus ruber	Red nucleus	7, 8	NAV^5th^; Welento, 2002
NRh	Nuclei rhaphe	Raphe nuclei	9	Butler and Hodos, 2005
NTSNT	Nucleus tractus spinalis n. trigemini	Nucleus of spinal tract of trigeminal nerve	10, 11, 12	NAV^5th^; Welento, 2002
NV	Nervus trigeminus	Trigeminal nerve	1, 8	NAV^5th^; Welento, 2002
NVb	Nuclei vestibulares	Vestibular nuclei	10	NAV^5th^; Welento, 2002
NVI	Nervus abducens	Abducent nerve	1, 9, 10	NAV^5th^; Welento, 2002
NVII	Nervus facialis	Facial nerve	10	NAV^5th^; Welento, 2002
NVIII	Nervus vestibulocochlearis	Vestibulocochlear nerve	10	NAV^5th^; Welento, 2002
NXII	Nervus hypoglossus	Hypoglossal nerve	11	NAV^5th^; Welento, 2002
OC	*b*	Olfactory cortex	3, 4	Butler and Hodos, 2005
PCC	Pedunculus cerebellaris caudalis	Caudal cerebellar peduncle	10, 11	NAV^5th^; Welento, 2002
PCM	Pedunculus cerebellaris medius	Middle cerebellar peduncle	9	NAV^5th^; Welento, 2002
PCR	Pedunculus cerebellaris rostralis	Rostral cerebellar peduncle	9	NAV^5th^; Welento, 2002
PCVQ	Plexus chorioideus ventriculi quart	Choroid plexus of fourth ventricle	10, 11	NAV^5th^; Welento, 2002
PFc	Pedunculus flocculi	Flocullar peduncle	10	NAV^5th^; Welento, 2002
PfD	Paraflocculus dorsalis	Dorsal paraflocculus	1, 9, 10, 11, 12	NAV^5th^; Welento, 2002
PfV	Paraflocculus ventralis	Ventral paraflocculus	1, 9, 10	NAV^5th^; Welento, 2002
PO	Pedunculus olfactorius	Olfactory peduncle	1, 3	NAV^5th^; Welento, 2002
Po	Pons	Pons	1, 8, 9	NAV^5th^; Welento, 2002
Pu	Putamen	Putamen	4, 5, 6, 7	NAV^5th^; Welento, 2002
Pv	Pulvinar	Pulvinar	7	NAV^5th^; Welento, 2002
PVm	Pyramis vermis	Pyramid of vermis	1	NAV^5th^; Welento, 2002
Py	Pyramis medulla oblongatae	Pyramid of medulla oblongata	1, 10, 11	NAV^5th^; Welento, 2002
Rh	Rhaphe	Raphe	9	NAV^5th^; Welento, 2002
SA	Sulcus ansatus	Ansiform sulcus	1	NAV^5th^; Welento, 2002
Sb	Subiculum	Subiculum	6, 7, 8	NAV^5th^; Welento, 2002
SBa	Sulcus basilaris	Basilar sulcus	1	NAV^5th^; Welento, 2002
SC	Sulcus coronalis	Coronal sulcus	1, 3, 4, 5, 6	NAV^5th^; Welento, 2002
SCC	Sulcus corporis callosi	Groove of corpus callosum	4, 5, 6, 7, 8	NAV^5th^; Welento, 2002
SCi	Sulcus cinguli	Cingulate sulcus	4, 5, 6, 7, 8	Meyer, 1964
SCr	Sulcus cruciatus	Cruciate sulcus	1, 3, 4	NAV^5th^; Welento, 2002
SCrM	Sulcus cruciatus minor	Lesser cruciate sulcus	1, 5, 6	Meyer, 1964
SeC	Septum cellulare	Septum of endbrain	4, 5	NAV^5th^; Welento, 2002
SeP	Septum pellucidum	Septum pellucidum	*c*	NAV^5th^; Welento, 2002
SEsC	Sulcus ectosylvius caudalis	Caudal ectosylvian sulcus	*c*	NAV^5th^; Welento, 2002
SEsR	Sulcus ectosylvius rostralis	Rostral ectosylvian sulcus	*c*	NAV^5th^; Welento, 2002
SGC	Substantia grisea centralis	Central gray substance	8, 9	NAV^5th^; Welento, 2002
SM	Sulcus marginalis	Marginal sulcus	1, 7, 8	NAV^5th^; Welento, 2002
SN	Substantia nigra	Substantia nigra	7, 8	NAV^5th^; Welento, 2002
SOc	Sulcus occipitalis	Occipital sulcus	1, 9	NAV^5th^; Welento, 2002
SP	Sulcus parietalis	Parietal sulcus	1, 7, 8	Mettler and Goss, 1946
SPc	Sulcus postcruciatus	Postcrucial sulcus	1, 5, 6	NAV^5th^; Welento, 2002
SPr	Sulcus proreus	Prorean sulcus	1, 2	NAV^5th^; Welento, 2002
SPs	Sulcus praesylvius	Presylvian sulcus	1, 3, 4	NAV^5th^; Welento, 2002
SRLPC	Sulcus rhinalis lateralis pars caudalis	Lateral rhinal sulcus – caudal part	1, 6	NAV^5th^; Welento, 2002
SRLPR	Sulcus rhinalis lateralis pars rostralis	Lateral rhinal sulcus – rostral part	1, 2, 3, 4, 5	NAV^5th^; Welento, 2002
SRM	Sulcus rhinalis medialis	Medial rhinal sulcus	1	NAV^5th^; Welento, 2002
SSs	Sulcus suprasplenialis	Suprasplenial sulcus	7, 8, 9	NAV^5th^; Welento, 2002
SSsC	Sulcus suprasylvius caudalis	Caudal suprasylvian sulcus	1, 7	
SSsR	Sulcus suprasylvius rostralis	Rostral suprasylvian sulcus	1, 4, 5, 6, 7	NAV^5th^; Welento, 2002
TC	Tuber cinereum	Tuber cinereum	1, 6	NAV^5th^; Welento, 2002
Th	Thalamus	Thalamus	6, 7	NAV^5th^; Welento, 2002
TNG	Tuberculum nuclei gracilis	Gracile tubercle	11	NAV^5th^; Welento, 2002
TO	Tuberculum olfactorium	Olfactory tubercle	1, 4, 5	NAV^5th^; Welento, 2002
TOI	Tractus olfactorius intermedius	Intermediate olfactory tract	4, 5	NAV^5th^; Welento, 2002
TOL	Tractus olfactorius lateralis	Lateral olfactory tract	3, 4, 5	NAV^5th^; Welento, 2002
TOM	Tractus olfactorius medialis	Medial olfactory tract	4	NAV^5th^; Welento, 2002
TOp	Tractus opticus	Optic tract	6, 7	NAV^5th^; Welento, 2002
TPy	Tractus pyramidalis	Pyramidal tract	9, 10, 11	NAV^5th^; Welento, 2002
TSNT	Tractus spinalis n. trigemini	Spinal tract of trigeminal nerve	9, 10, 11, 12	NAV^5th^; Welento, 2002
TVm	Tuber vermis	Tuber of vermis	1, 12	NAV^5th^; Welento, 2002
UVm	Uvula vermis	Uvula vermis	1, 11, 12	NAV^5th^; Welento, 2002
VL	Ventriculus lateralis	Lateral ventricle	4, 5, 6, 7, 8	NAV^5th^; Welento, 2002
Vm	Vermis	Vermis	1, 9, 10, 11, 12	NAV^5th^; Welento, 2002
VMR	Velum medullare rostrale	Rostral medullary velum	9	NAV^5th^; Welento, 2002
VQ	Ventriculus quartus	Fourth ventricle	9, 10, 11	NAV^5th^; Welento, 2002
VT	Ventriculus tertius	Third ventricle	6, 7	NAV^5th^; Welento, 2002
*a*	Cerebellum	Cerebellum	1, 9, 10, 11, 12	NAV^5th^; Welento, 2002
*a*	Diencephalon	Diencephalon	1, 6, 7	NAV^5th^; Welento, 2002
*a*	Fossa rhomboidea	Rhomboid fossa	9, 10, 11	NAV^5th^; Welento, 2002
*a*	Genu corporis callosi	Genu of corpus callosum	4	NAV^5th^; Welento, 2002
*a*	Gyrus praecruciatus	Precruciate gyrus	*c*	NAV^5th^; Welento, 2002
*a*	Gyrus sylvius caudalis	Caudal sylvian gyrus	*c*	NAV^5th^; Welento, 2002
*a*	Gyrus sylvius rostralis	Rostral sylvian gyrus	*c*	NAV^5th^; Welento, 2002
*a*	Hypophysis = glandula pituitaria	Hypophysis = pituitary gland	*d*	NAV^5th^; Welento, 2002
*a*	Hypothalamus	Hypothalamus	1, 6	NAV^5th^; Welento, 2002
*a*	Lamina medullaris medialis	Medial medullary lamina	*c*	NAV^5th^; Welento, 2002
*a*	Mesencephalon	Midbrain	7, 8, 9	NAV^5th^; Welento, 2002
*a*	Nervus opticus	Optic nerve	1	NAV^5th^; Welento, 2002
*a*	Nucleus lentiformis	Lentiform nucleus	4, 5, 6, 7	NAV^5th^; Welento, 2002
*a*	Nucleus motorius n. hypoglossi	Motor nucleus of hypoglossal nerve	*e*	NAV^5th^; Welento, 2002
*a*	Nucleus parasympathicus n. vagi	Parasympathetic nucleus of vagus nerve	*e*	NAV^5th^; Welento, 2002
*a*	Nucleus tractus solitarii	Solitary nucleus	*e*	NAV^5th^; Welento, 2002
*a*	Pallium dorsale	Dorsal pallium	1, 2, 3, 4, 5, 6, 7, 8, 9	Butler and Hodos, 2005
*a*	Pallium laterale	Lateral pallium	1, 3, 4, 5, 6, 7	Butler and Hodos, 2005
*a*	Pallium mediale	Medial pallium	6, 7, 8	Butler and Hodos, 2005
*a*	Pars dorsalis pontis	Dorsal part of pons	8, 9	NAV^5th^; Welento, 2002
*a*	Pars ventralis pontis	Ventral part of pons	8, 9	NAV^5th^; Welento, 2002
*a*	Rhinencephalon	Olfactory brain	1, 2, 3, 4, 5	NAV^5th^; Welento, 2002
*a*	Striatum	Striatum	4, 5, 6, 7	Butler and Hodos, 2005
*a*	Subthalamus	Subthalamus	6, 7	NAV^5th^; Welento, 2002
*a*	Sulcus endomarginalis	Endomarginal sulcus	*c*	NAV^5th^; Welento, 2002
*a*	Sulcus suprasylvius medius	Middle suprasylvian sulcus	*c*	NAV^5th^; Welento, 2002
*a*	Tectum mesencephali	Tectum of midbrain	8, 9	NAV^5th^; Welento, 2002
*a*	Tegmentum mesencephali	Tegmentum of midbrain	7, 8, 9	NAV^5th^; Welento, 2002
*a*	Telencephalon	Telencephalon endbrain	1, 2, 3, 4, 5, 6, 7, 8, 9	NAV^5th^; Welento, 2002
*a*	Zona incerta	Uncertain zone	*e*	NAV^5th^; Welento, 2002

*The list of figures of the structures without abbreviation but present in *U. arctos* is also included but the structures are not pinpointed in the figures, for example the telencephalon. *a*, abbreviation not used; *b*, term absent in NAV^5*th*^ or other references. *c*, structure absent in *U. arctos* but mentioned in the paper. *d*, structure present in *U. arctos* but not captured in the figures. *e*, structure probably present in *U. arctos* but not identified.*

## Results

### Telencephalon – Gross Anatomy Including Sulci and Gyri Pattern

**FIGURE 1:**
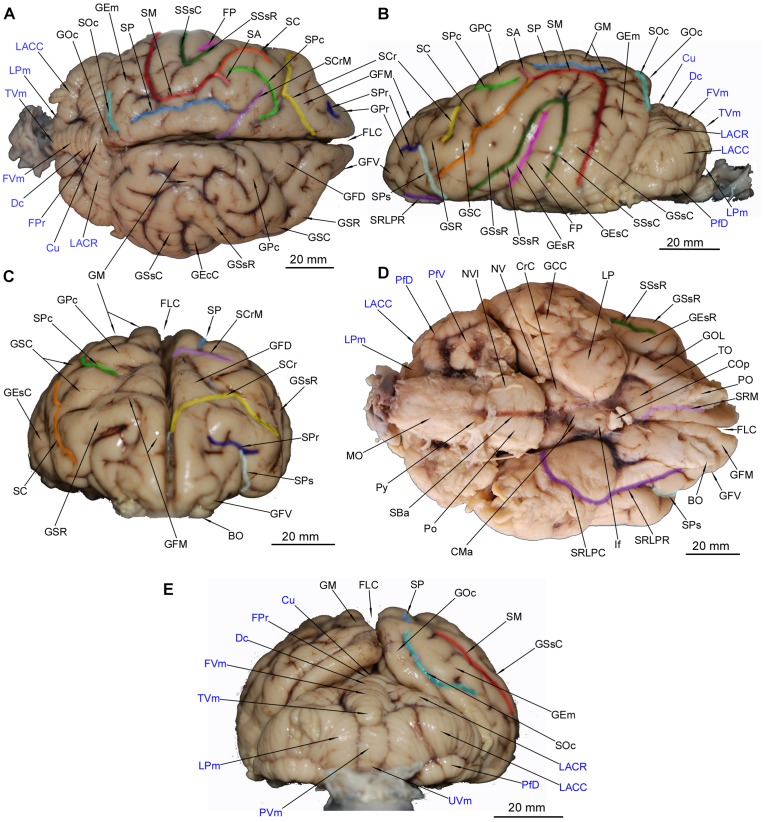
Gross anatomy of the *Ursus arctos* brain. **(A)** [top, left] dorsal surface. **(B)** [top, right] lateral surface. **(C)** [middle left] anterior surface. **(D)** [middle right] ventral surface. **(E)** [bottom] posterior surface. The cerebellar structures were written in blue color. Each sulcus was marked in different color that indicates its course visible from given perspective. BO, bulbus olfactorius; CMa, corpus mamillare; COp, chiasma opticum; CrC, crus cerebri; Cu, culmen; Dc, declive; FLC, fissura longitudinalis cerebri; FP, fissura pseudosylvia; FPr, fissura prima; FVm, folium vermis; GCC, gyrus compositus caudalis; GEm, gyrus ectomarginalis; GEsC, gyrus ectosylvius caudalis; GEsR, gyrus ectosylvius rostralis; GFD, gyrus frontalis dorsalis; GFM, gyrus frontalis medius; GFV, gyrus frontalis ventralis; GM, gyrus marginalis; GOc, gyrus occipitalis; GOL, gyrus olfactorius lateralis; GPc, gyrus postcruciatus; GPr, gyrus proreus; GSC, gyrus sigmoideus caudalis; GSR, gyrus sigmoideus rostralis; GSsC, gyrus suprasylvius caudalis; GSsR, gyrus suprasylvius rostralis; If, infundibulum; LACC, lobulus ansiformis, crus caudale; LACR, lobulus ansiformis, crus rostrale; LP, lobus piriformis; LPm, lobulus paramedianus; MO, medulla oblongata; NV, nervus trigeminus; NVI, nervus abducens; PfD, paraflocculus dorsalis; PfV, paraflocculus ventralis; PO, pedunculus olfactorius; Po, pons; PVm, pyramis vermis; Py, pyramis medulla oblongatae; SA, sulcus ansatus; SBa, sulcus basilaris; SC, sulcus coronalis; SCr, sulcus cruciatus; SCrM, sulcus cruciatus minor; SM, sulcus marginalis; SOc, sulcus occipitalis; SP, sulcus parietalis; SPc, sulcus postcruciatus; SPr, sulcus proreus; SPs, sulcus praesylvius; SRLPC, sulcus rhinalis lateralis, pars caudalis; SRLPR, sulcus rhinalis lateralis, pars rostralis; SRM, sulcus rhinalis medialis; SSsC, sulcus suprasylvius caudalis; SSsR, sulcus suprasylvius rostralis; TO, tuberculum olfactorium; TVm, tuber vermis; UVm, uvula vermis.

The brain surface of the brown bear is highly convoluted, suggesting an elaborate underlying structure. The most medially located structure is the gyrus marginalis (GM) (Figure 1A). GM is divided by the sulcus parietalis (SP) into two parts – a smaller medial and larger lateral part. However, we found that the trait varies – in another individual (RH 155/14) only unilateral division was present and in another one (RH 194/14) the gyrus was not divided at all.

GM continues rostrally as a short gyrus postcruciatus (GPc) and then two winding structures- gyrus sigmoideus caudalis (GSC) and gyrus sigmoideus rostralis (GSR). The border between the latter two is the sulcus cruciatus indentation. GM is bordered laterally by sulcus marginalis (SM) with its caudal part bending to the lateral surface of the hemisphere (Figure 1B) and a shorter rostral part continued as the sulcus coronalis (SC) intercepting a short sulcus ansatus (SA).

Gyrus marginalis continues caudally with an apparent narrowing where the gyrus occipitalis (GOc) is bordered laterally by the sulcus occipitalis (SOc) (Figure 1E). The gyrus ectomarginalis (GEm) runs parallel to fissura pseudosylvia (FP) where its rostral section borders with it (Figure 1A). Three sulci on the dorsal aspect take a transverse position. The most rostral is the sulcus cruciatus (SCr) dividing the gyrus frontalis dorsalis (GFD) from the gyrus frontalis medius (GFM). More caudally but close to SCr runs sulcus postcruciatus (SPc) separating GFD from GPc (Figure 1A). Both abovementioned sulci are also visible from the lateral aspect (Figure 1B) and are surrounded by GSR and GSC. The third sulcus is the sulcus cruciatus minor (SCrM) running rostro-laterally separating GM and GSC from a rostrally lying GPc (Figure 1A). On the lateral aspect a deep but very tight FP separates the gyrus ectosylvius rostralis (GEsR) from the gyrus ectosylvius caudalis (GEsC) – those gyri are separated from overlying gyrus suprasylvius rostralis (GSsR) and gyrus suprasylvius caudalis (GSsR) by sulcus suprasylvius rostralis (SSsR) and sulcus suprasylvius caudalis (SSsC) respectively. The abovementioned gyri and sulci converge at an acute angle that constitutes the border between rostral and lateral gyri and sulci on the lateral aspect (Figure 1B). The rostral pole of the cerebral hemisphere is occupied mainly by the gyri frontales (Figure 1C).

The most signature structure, a very distinctive lozenge, is formed by the GFD with the contralateral gyrus. The horizontal part of GFM runs medially until its vertical part running to the pole of the hemisphere, eventually becoming the ventral aspect of the brain (Figures 1C,D). The gyrus frontalis ventralis (GFV) lies more ventrally and runs horizontally converging with GFM and bordering with the gyrus proreus (GPr) dorsally. GPr is separated from a dorsally lying GSR by the sulcus proreus (SPr) with the sulcus praesylvius (SPs) converging with it (Figure 1C).

The main external structures of the rhinencephalon are easily recognized in the ventral aspect of the brain (Figure 1D). The bulbus olfactorius (BO) is present only as its caudal parts in the investigated specimen. The pedunculus olfactorius (PO) is apparent and continued caudally as the tractus olfactorius – lateralis (TOL), medialis (TOM) and intermedius (TOI). The gyrus olfactorius lateralis (GOL) is separated laterally from the dorsal pallium gyri by the sulcus rhinalis lateralis – pars rostralis (SRLPR). The sulcus is continued caudally as its pars caudalis (SRLPC) and separates lobus piriformis (LP) from caudo-laterally lying parts of the temporal lobe with the dorsal pallium derivatives. A round and distinctive tuberculum olfactorium (TO) lies just in the front of LO and medially from GOL. The remaining area of the ventral surface of the telencephalon is occupied by the dorsal pallium. The caudal surface is occupied by the gyrus compositus caudalis (GCC) as a result of GEsC, GSsC and GEm ventral convergence. Rostrally, GFM is separated from PO by the sulcus rhinalis medialis (SRM). Also a small part of GFV is noticeable laterally to PO.

**FIGURE 2:**
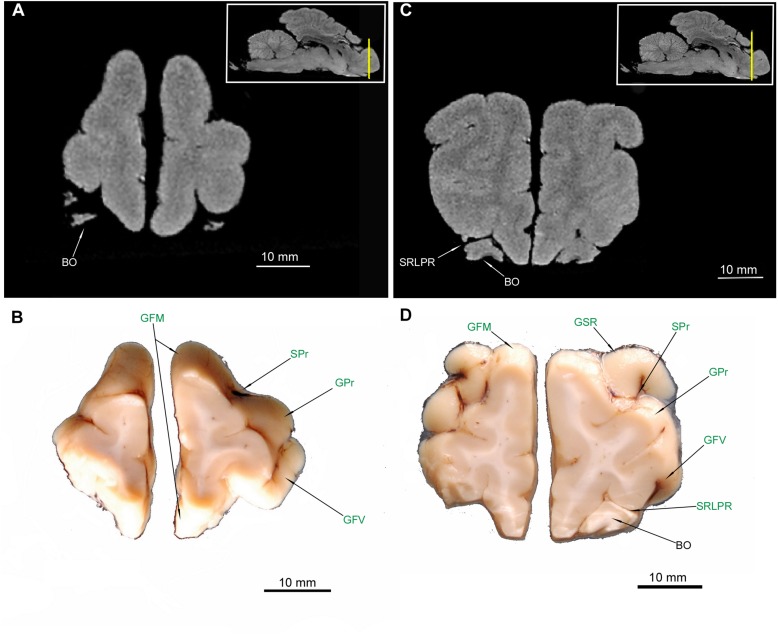
MRI imaging and cross sections and through the most anterior part of the telencephalon in the area of gyri frontales and gyrus proreus in *U. arctos.* **(A,B)** MRI and macroscopic anatomy cross-section through the anterior pole of the cerebral hemispheres. **(C,D)** MRI and macroscopic anatomy cross-section through anterior telencephalon with the pair of bulbus olfactorius included in MRI. The lack of right bulbus olfactorius in **(D)** is an artifact. The insets in **(A,C)** show the exact plane of cross-section in MRI and the best achieved correspondence in macroscopic anatomy **(B,D)**. In **(B,D)** gyri and sulci of the telencephalon were written in green color. BO, bulbus olfactorius; GFM, gyrus frontalis medius; GFV, gyrus frontalis ventralis; GPr, gyrus proreus; GSR, gyrus sigmoideus rostralis; SRLPR, sulcus rhinalis lateralis, pars rostralis; SPr, sulcus proreus.

All of the abovementioned gyri, sulci and the rhinencephalon structures were confirmed in the cross-sectional anatomical images. The MRI images appeared devoid of sulci and gyri- only rhinencephalic structures showed good correspondence with macroscopic anatomy cross-sections. The lack of right BO in Figure 2D is caused by preparation defect, however, it is noticeable bilaterally in MRI (Figures 2A,C). Furthermore, in cross sections of the medial aspect of the hemispheres, the sulcus cinguli (SCi) was depicted separating the gyrus cinguli (GCi) from an above-lying GFD in the rostral part (Figures 4D, 5B,D, 6B), from GM in the middle part (Figures 6D, 7B) and from the gyrus splenialis (GS) in the caudal part (Figures 7D, 8B,D, 9B). The sulcus corporis callosi (SCC) borders GCi ventrally. GS is bordered dorsally by the sulcus suprasplenialis (SSs) (Figures 7B,D, 8B,D, 9B).

### Telencephalon – Internal Anatomy

The putamen (Pu) is distinctive in the macroscopic anatomical cross-sections (MAC) and the MRI cross-sections (MRI) (Figures 4–6, 7B). The globus pallidus (GP) (Figures 4C,D, 5, 6) is primarily visible in MAC; in MRI it is depicted together with Pu. The claustrum (Cm) (Figures 4–6, 7A,B,D) and all parts of the nucleus caudatus (NC) were also identified (Figures 4–6, 7B). The latter is always present in the vicinity of the ventriculus lateralis (VL) (Figures 4–7, 8A–C). The nucleus accumbens (NAc) (Figures 5C,D) and the corpus amygdaloideum (CAg) (Figure 6) are also visible. The septum cellulare (SeC) is apparent due to specific topography (Figures 4D, 5) but the septum pellucidum (SeP) is absent, despite thorough tracing of each cross-section across the brain. The hippocampal formation with all of its major subdivision was depicted in both types of cross-sections (Figures 6C,D, 7, 8A,B).

The most prominent white matter (in MRI hypointense) was also depicted – all parts of the corpus callosum (CC) (Figures 4–7, 8B) and all parts of the fornix (Fx) (Figures 6, 7). The capsula interna (CI) is visible both in MAC and MRI (Figures 4–6, 7A,B,D). The capsula externa (CE) was primarily visualized in MAC whereas in MRI was noticeable only in the one cross-section (Figures 4D, 5B,D, 6A,B,D, 7B). The same goes for the capsula extrema (CEx) – distinct in MAC, but visualized only in two MRI cross-sections (Figures 4A,B,D, 5A,B,D, 6B,D, 7B,D).

**FIGURE 3:**
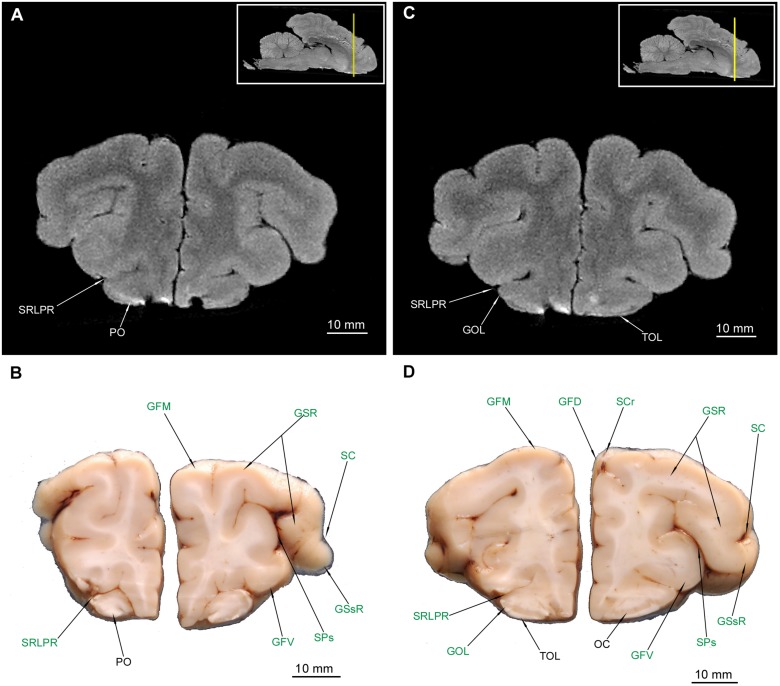
MRI imaging and cross sections through the anterior part of telencephalon in the area of gyri frontales and pedunculus olfactorius – tractus olfactorius interface in *U. arctos.* **(A,B)** MRI and macroscopic anatomy cross-section through the anterior area of the cerebral hemispheres with pedunculi olfactorii. Note in **(A)** that in MRI the signal from the pedunculi is similar to that of gyri. **(C,D)** MRI and macroscopic anatomy cross-section through the anterior part of cerebral hemispheres just before the inception of ventriculus lateralis lumen. The insets in **(A,C)** shows the exact plane of cross-section in MRI and the best achieved correspondence in macroscopic anatomy **(B,D)**. In **(B,D)** gyri and sulci of the telencephalon were written in green color. GFD, gyrus frontalis dorsalis; GFM, gyrus frontalis medius; GFV, gyrus frontalis ventralis; GOL, gyrus olfactorius lateralis; GSR, gyrus sigmoideus rostralis; GSsR, gyrus suprasylvius rostralis; OC, olfactory cortex; PO, pedunculus olfactorius; SC, sulcus coronalis; SCr, sulcus cruciatus; SPs, sulcus praesylvius; SRLPR, sulcus rhinalis lateralis, pars rostralis; TOL, tractus olfactorius lateralis.

**FIGURE 4:**
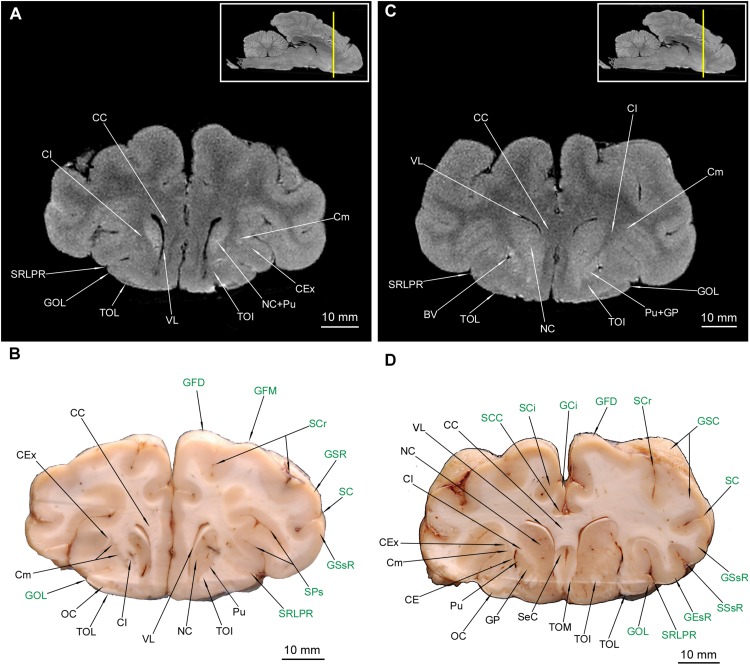
MRI imaging and cross sections through the anterior part of telencephalon in the area of corpus callosum, claustrum and striatum inception in *U. arctos.* **(A,B)** MRI and macroscopic anatomy cross-section through the anterior area of the cerebral hemispheres with beginning of putamen, nucleus caudatus, claustrum and corpus callosum. Note strong signal from putamen-nucleus caudatus in **(A)**. **(C,D)** MRI and macroscopic anatomy cross-section through the anterior area of the cerebral hemispheres interconnected by the corpus collosum and with beginning of gray matter of the septum cellulare. Note the blood vessels in the putamen-capsula externa interface in **(D)** and characteristic lack of signal in spots of blood vessels occurrence in MRI in **(C)**. The insets in **(A,C)** shows the exact plane of cross-section in MRI and the best achieved correspondence in macroscopic anatomy **(B,D)**. In **(B,D)** gyri and sulci of the telencephalon were written in green color. BV, blood vessel; CC, corpus callosum; CE, capsula externa; CEx, capsula extrema; CI, capsula interna; Cm, claustrum; NC, nucleus caudatus; GCi, gyrus cinguli; GEsR, gyrus ectosylvius rostralis; GFD, gyrus frontalis dorsalis; GFM, gyrus frontalis medius; GOL, gyrus olfactorius lateralis; GP, globus pallidus; GSC, gyrus sigmoideus caudalis; GSR, gyrus sigmoideus rostralis; GSsR, gyrus suprasylvius rostralis; OC, olfactory cortex; Pu, putamen; SC, sulcus coronalis; SCi, sulcus cinguli; SCC, sulcus corporis callosi; SCr, sulcus cruciatus; SeC, septum cellulare; SPs, sulcus praesylvius; SRLPR, sulcus rhinalis lateralis, pars rostralis; SSsR, sulcus suprasylvius rostralis; TOI, tractus olfactorius intermedius; TOL, tractus olfactorius lateralis; TOM, tractus olfactorius medialis; VL, ventriculus lateralis.

**FIGURE 5:**
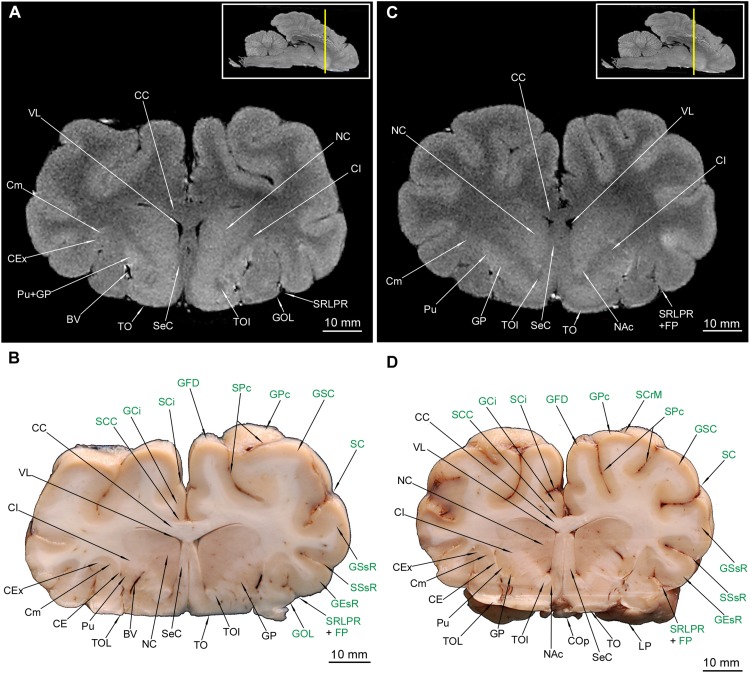
MRI imaging and cross sections through the middle part of telencephalon in the area of the septum cellulare, nucleus accumbens and expansion of the nucleus caudatus in *U. arctos.* **(A,B)** MRI and macroscopic anatomy cross-section through the middle area of the cerebral hemispheres with the expansion of septum cellulare and nucleus caudatus. Note blood vessel between prominent striatal structures in **(B)**. **(C,D)** MRI and macroscopic anatomy cross-section through the middle part of cerebral hemispheres. Note excellent visibility of the nucleus accumbens in MRI **(C)** and highly delineated lentoid in shape putamen in **(D)**. The insets in **(A,C)** shows the exact plane of cross-section in MRI and the best achieved correspondence in macroscopic anatomy **(B,D)**. In **(B,D)** gyri and sulci of the telencephalon were written in green color. BV, blood vessel; CC, corpus callosum; CE, capsula externa; CEx, capsula extrema; CI, capsula interna; Cm, claustrum; COp, chiasma opticum; FP, fissura pseudosylvia; GCi, gyrus cinguli; GEsR, gyrus ectosylvius rostralis; GFD, gyrus frontalis dorsalis; GOL, gyrus olfactorius lateralis; GP, globus pallidus; GSC, gyrus sigmoideus caudalis; GSsR, gyrus suprasylvius rostralis; LP, lobus piriformis; NAc, nucleus accumbens; NC, nucleus caudatus; Pu, putamen; SC, sulcus coronalis; SCC, sulcus corporis callosi; SCi, sulcus cinguli; SCrM, sulcus cruciatus minor; SeC, septum cellulare; SPc, sulcus postcruciatus; SRLPR, sulcus rhinalis lateralis, pars rostralis; SSsR, sulcus suprasylvius rostralis; TO, tuberculum olfactorium; TOI, tractus olfactorius intermedius; TOL, tractus olfactorius lateralis; VL, ventriculus lateralis.

TOL (Figures 3C,D, 4, 5B,D) and TOI (Figures 4, 5) were depicted in both MAC and MRI. However TOM proved to be very thin and was detected only in MAC (Figure 4D). The olfactory cortex (OC) is visible in Figures 3D, 4B,D.

### Diencephalon

**FIGURE 6:**
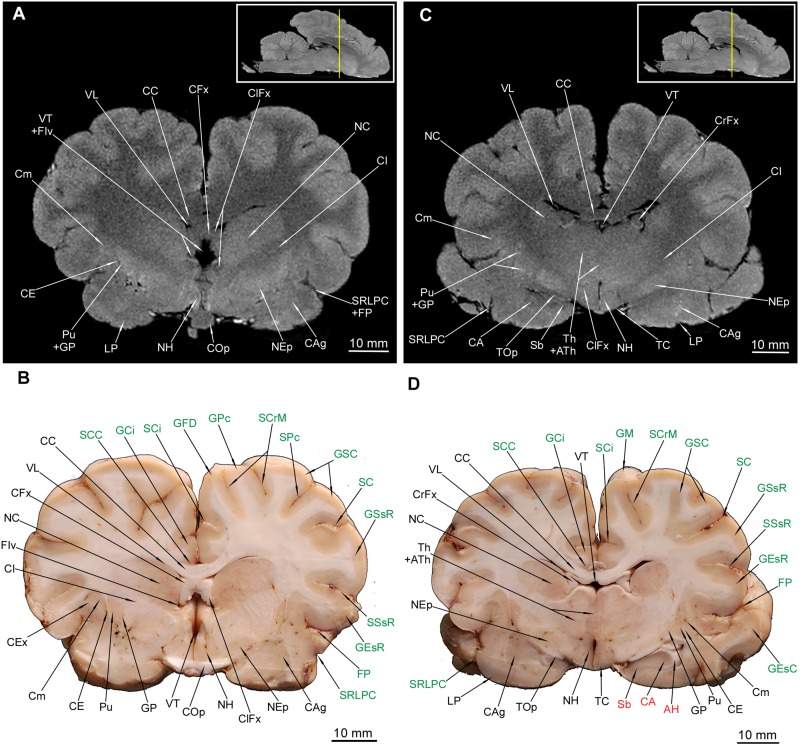
MRI imaging and cross sections through the middle part of telencephalon in the area of the corpus amygdaloideum, nucleus endopeduncularis and the inception of hippocampal formation and through the diencephalon with the thalamus and hypothalamus in *U. arctos.* **(A,B)** MRI and macroscopic anatomy cross-section through the middle area of the cerebral hemispheres and inception of the hypothalamus. Note the amygdala with its mixed signal in MRI **(A)** and interlacing white and gray matter in **(B)** and the distinct nucleus endopenduncularis in **(A)**. Gray matter of hypothalamus begins in this cross section **(A,B)**. Intensively hypointense middle “bulge” **(A)** is the chiasma opticum. **(C,D)** MRI and macroscopic anatomy cross-section through the middle part of cerebral hemisphere and the inception of thalamus. Note reduction of the nucleus caudatus diameter in **(D)** and its signal decrement in MRI **(C)**; the ventriculus tertius is extremely reduced by the adhaesio interthalamica. Due to slight asymmetry in **(D)** the right corpus amygdaloideum and the left hippocampal formation were captured. The insets in **(A,C)** shows the exact plane of cross-section in MRI and the best achieved correspondence in macroscopic anatomy **(B,D)**. In **(B,D)** gyri and sulci of the telencephalon were written in green color and structures of hippocampal formation in red color. AH, alveus hippocampi; ATh, adhaesio interthalamica; CA, cornu ammonis; CAg, corpus amygdaloideum; CC, corpus callosum; CE, capsula externa; CEx, capsula extrema; CFx, corpus fornicis; CI, capsula interna; ClFx, columna fornicis; Cm, claustrum; COp, chiasma opticum; CrFx, crus fornicis; GCi, gyrus cinguli; GEsC, gyrus ectosylvius caudalis; GEsR, gyrus ectosylvius rostralis; GFD, gyrus frontalis dorsalis; GM, gyrus marginalis; GP, globus pallidus; GPc, gyrus postcruciatus; GSC, gyrus sigmoideus caudalis; GSsR, gyrus suprasylvius rostralis; FIv, foramen interventriculare; FP, fissura pseudosylvia; LP, lobus piriformis; NC, nucleus caudatus; NEp, nucleus endopeduncularis; NH, nuclei hypothalamici; Pu, putamen; Sb, subiculum; SC, sulcus coronalis; SCC, sulcus corporis callosi; SCi, sulcus cinguli; SCrM, sulcus cruciatus minor; SPc, sulcus postcruciatus; SRLPC, sulcus rhinalis lateralis, pars caudalis; SSsR, sulcus suprasylvius rostralis; TC, tuber cinereum; Th, thalamus; TOp, tractus opticus; VT, ventriculus tertius; VL, ventriculus lateralis.

Out of diencephalic external structures (Figure 1D) the following were depicted rostral-caudal: the chiasma opticum (COp) (Figures 5D, 6A,B) with a visible section of the nervus opticus (NII), an unpaired infundibulum (If) (the hypophysis was not preserved during the preparation), an unpaired tuber cinereum (TC) and a pair of corpora mammillaria (CMa).

**FIGURE 7:**
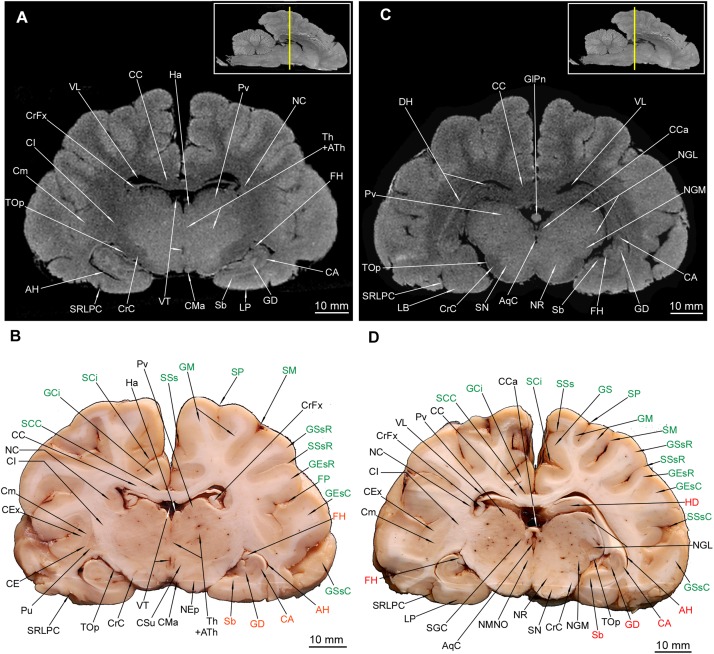
MRI imaging and cross sections through the middle and caudal parts of telencephalon with the hippocampal formation, through the full extension of the diencephalon and inception of the mesencephalon in *U. arctos.* **(A,B)** MRI and macroscopic anatomy cross-section through the middle area of the cerebral hemispheres with the hippocampal formation. The right putamen is still noticeable in **(B)** due to a slight asymmetry. Note excellent resolution of the hippocampal formation components in MRI **(A)** comparable with that in **(B)**. The nucleus caudatus is still more reduced in diameter and nearly indiscernible in MRI. **(C,D)** MRI and macroscopic anatomy cross-section through the caudal part of cerebral hemisphere and the inception of mesencephalon. Note the curvature of the hippocampal formation in **(C,D)**. The claustrum is still distinct in **(D)** due to a slight asymmetry. Note the upper and lower parts of the nucleus geniculatus lateralis, the beginning of substantia nigra and nucleus ruber. The insets in **(A,C)** shows the exact plane of cross-section in MRI and the best achieved correspondence in macroscopic anatomy **(B,D)**. In **(B,D)** gyri and sulci of the telencephalon were written in green color and structures of hippocampal formation in red color. AH, alveus hippocampi; AqC, aquaeductus cerebri; ATh, adhaesio interthalamica; CA, cornu ammonis; CC, corpus callosum; CCa, commissura caudalis; CE, capsula externa; CEx, capsula extrema; CI, capsula interna; Cm, claustrum; CMa, corpus mamillare; CrC, crus cerebri; CrFx, crus fornicis; CSu, corpus subthalamicum; GCi, gyrus cinguli; GD, gyrus dentatus; GEsC, gyrus ectosylvius caudalis; GEsR, gyrus ectosylvius rostralis; GlPn, glandula pinealis; GM, gyrus marginalis; GS, gyrus splenialis; GSsC, gyrus suprasylvius caudalis; GSsR, gyrus suprasylvius rostralis; Ha, habenula; HD, dorsal hippocampal formation; FH, fimbria hippocampi; FP, fissura pseudosylvia; LP, lobus piriformis; NC, nucleus caudatus; NEp, nucleus endopeduncularis; NGL, nucleus geniculatus lateralis; NGM, nucleus geniculatus medialis; NMNO, nucleus motorius n. oculomotorii; NR, nucleus ruber; Pu, putamen; PV, pulvinar; Sb, subiculum; SCC, sulcus corporis callosi; SCi, sulcus cinguli; SGC, substantia grisea centralis; SM, sulcus marginalis; SN, substantia nigra; SP, sulcus parietalis; SRLPC, sulcus rhinalis lateralis, pars caudalis; SSs, sulcus suprasplenialis; SSsR, sulcus suprasylvius rostralis; Th, thalamus; TOp, tractus opticus; VT, ventriculus tertius; VL, ventriculus lateralis.

**FIGURE 8:**
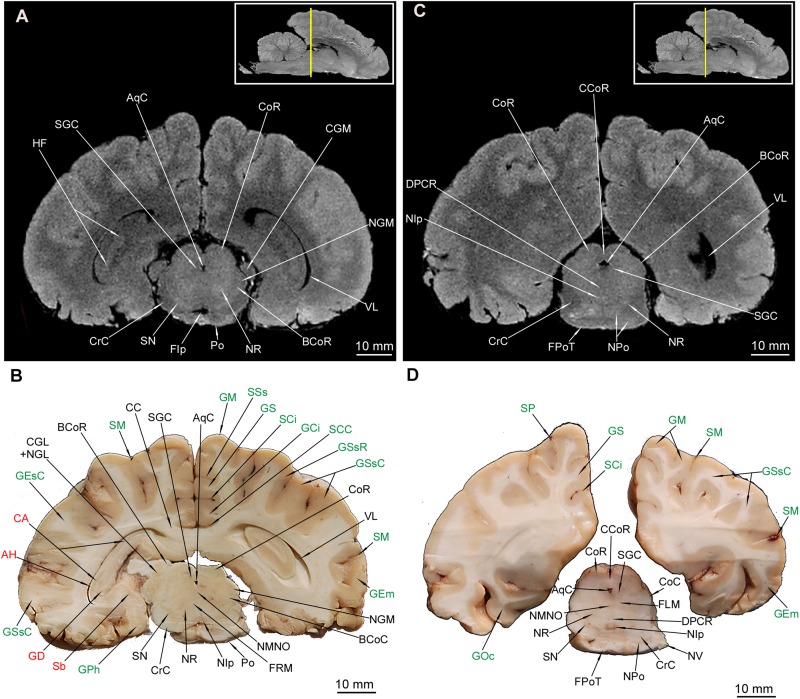
MRI imaging and cross sections through the caudal part of telencephalon with the closing of hippocampal formation, through the full extension of mesencephalon and inception of the pars ventralis pontis in *U. arctos.* **(A,B)** MRI and macroscopic anatomy cross-section through the caudal area of cerebral hemispheres with the caudal part of the hippocampal formation curvature, the mesencephalon and the most rostral part of the pons. The hippocampal tissue is still discernible both in **(A,B)**. The nucleus ruber and substantia nigra assume their full expansion in these cross-sections. Note visible stratification of colliculus rostralis in MRI **(A)**. **(C,D)** MRI and macroscopic anatomy cross-section through the caudal part of no longer interconnected cerebral hemispheres, the distal area of the mesencephalon and the rostral area of pars ventralis pontis. Note the very distinct nucleus interpeduncularis as well as the closing parts of the nucleus ruber and substantia nigra **(D)**. A distinct separation of pontine and mesencephalic tissue is evident in MRI **(C)** as hypointense stripes. The insets in **(A,C)** shows the exact plane of cross-section in MRI and the best achieved correspondence in macroscopic anatomy **(B,D)**. In **(B,D)** gyri and sulci of the telencephalon were written in green color and structures of hippocampal formation in red color. AH, alveus hippocampi; AqC, aquaeductus cerebri; BCoC, brachium colliculi caudalis; BCoR, brachium colliculi rostralis; CA, cornu ammonis; CC, corpus callosum; CCoR, commissura colliculorum rostralium; CGL, corpus geniculatum laterale; CGM, corpus geniculatum mediale; CoC, colliculus caudalis; CoR, colliculus rostralis; CrC, crus cerebri; DPCR, decussatio pedunculorum cerebellarium rostralium; FIp, fossa interpeduncularis; FLM, fasciculus longitudinalis medialis; FPoT, fibrae pontis transversae; FRM, formatio reticularis mesencephali; GCi, gyrus cinguli; GD, gyrus dentatus; GEm, gyrus ectomarginalis; GEsC, gyrus ectosylvius caudalis; GM, gyrus marginalis; GOc, gyrus occipitalis; GPh, gyrus parahippocampalis; GS, gyrus splenialis; GSsC, gyrus suprasylvius caudalis; GSsR, gyrus suprasylvius rostralis; HF, hippocampal formation; NGL, nucleus geniculatus lateralis; NGM, nucleus geniculatus medialis; NIp, nucleus interpeduncularis; NMNO, nucleus motorius n. oculomotorii; NPo, nuclei pontis; NR, nucleus ruber; NV, nervus trigeminus; Po, pons; Sb, subiculum; SCC, sulcus corporis callosi; SCi, sulcus cinguli; SGC, substantia grisea centralis; SM, sulcus marginalis; SN, substantia nigra; SP, sulcus parietalis; SSs, sulcus suprasplenialis; VL, ventriculus lateralis.

The thalamus (Th) with countable blood vessels was depicted as a broad centrally located gray matter or a hyperintense area (Figures 6C,D, 7). However, none of the hypothalamic nuclei were apparent, with the exception of the most posterior region – the pulvinar (Pv) (Figure 7). The corpus subthalamicum (CSu) was only depicted in MAC (Figure 7B) and the nucleus endopeduncularis (NEp) in both methods (Figures 6, 7A,B). The zona incerta was not identifiable in any of the cross-sections. The proper hypothalamus – nuclei hypothalamici (NH) is also depicted (Figure 6) but none of the particular nuclei were identifiable. The gray matter of superficial structures – TC (Figures 6C,D) and CMa was also visible in the cross sections (Figures 7A,B). Tractus opticus (TOp) (Figures 6C,D, 7) were also depicted. Both the corpora geniculata – corpus geniculatum mediale (CGM) et laterale (CGL) with their nuclei were identifiable in both methods (Figures 7C,D, 8A,B). The glandula pinealis (GlPn) is depicted only in MRI as highly hyperintense round area (Figure 7C). The habenulae (Ha) were identified with both methods (Figures 7A,B) as well as the comissura caudalis (CCa) - above the ventriculus tertius (VT) – aquaeductus cerebri (AqC) border (Figures 7C,D).

### Mesencephalon

**FIGURE 9:**
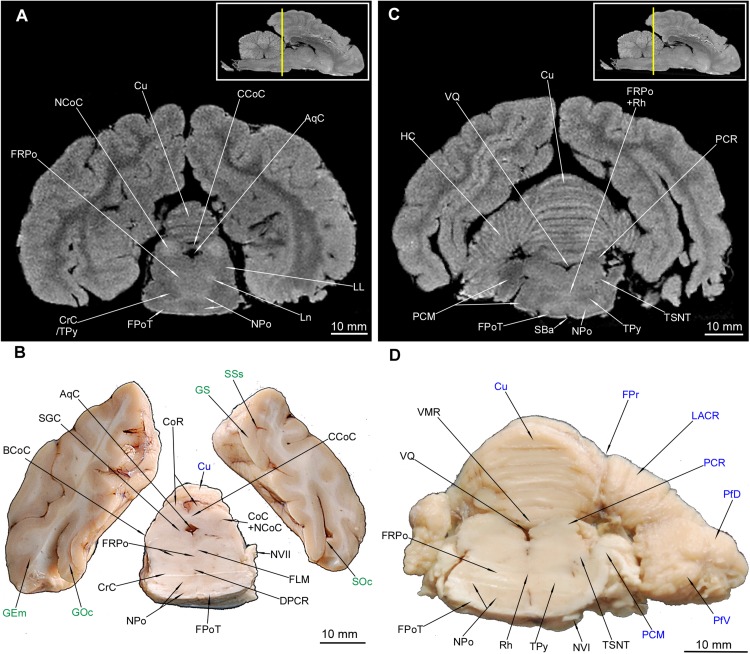
MRI imaging and cross sections through the most caudal parts of telencephalon and mesencephalon, through the full extension of pons and the inception of cerebellum in *U. arctos.* **(A,B)** MRI and macroscopic anatomy cross-section through the most caudal area of cerebral hemispheres, mesencephalon and pars ventralis pontis. Subcortical structures are no longer visible. Note the very distinct nucleus colliculi caudalis in MRI **(A)** and the initial fragment of the vermis in **(A, B)**. **(C,D)** MRI and macroscopic anatomy cross-section through the pons and initial fragment of the fossa rhomboidea and cerebellar hemispheres. Note very good distinction between white and gray matter of pons in MRI **(C)** and the pedunculus cerebellaris medius – fibrae pontis transverse transition in **(D)**. The insets in **(A,C)** shows the exact plane of cross-section in MRI and the best achieved correspondence in macroscopic anatomy **(B,D)**. In **(B,D)** gyri and sulci of the telencephalon were written in green color and the cerebellar structures were written in blue color. AqC, aquaeductus cerebri; BCoC, brachium colliculi caudalis; CCoC, commissura colliculorum caudalium; CoC, colliculus caudalis; CoR, colliculus rostralis; CrC, crus cerebri; Cu, culmen; DPCR, decussatio pedunculorum cerebellarium rostralium; FLM, fasciculus longitudinalis medialis; FPoT, fibrae pontis transversae; FPr, fissura prima; FRPo, formatio reticularis pontis; GEm, gyrus ectomarginalis; GOc, gyrus occipitalis; GS, gyrus splenialis; HC, hemisphaerium cerebelli; LACR, lobulus ansiformis, crus rostrale; LL, lemniscus lateralis; Ln, lesion; NCoC, nucleus colliculi caudalis; NPo, nuclei pontis; NVI, nervus abducens; NVII, nervus facialis; PCM, pedunculus cerebellaris medius; PCR, pedunculus cerebellaris rostralis; PfD, paraflocculus dorsalis; PfV, paraflocculus ventralis; Rh, rhaphe; SBa, sulcus basilaris; SGC, substantia grisea centralis; SOc, sulcus occipitalis; SSs, sulcus suprasplenialis; TPy, tractus pyramidalis; TSNT, tractus spinalis n. trigemini; VMR, velum medullare rostrale; VQ, ventriculus quartus.

Out of external mesencephalic structures a pair of crura cerebri (CrC) was visualized with a distinct dimple between them – the fossa interpeduncularis (FIp) (Figure 1D). Elements of the tectum were depicted. Colliculus rostralis (CoR) (Figures 8, 9B) and colliculus caudalis (CoC) (Figures 8D, 9A,B) with the former evident as a striated organization in MRI and the latter depicted with its nuclei – the nucleus colliculi caudalis (NCoC) (Figures 9A,B). The commissura colliculorum caudalium (CCoC) (Figures 9A,B) et colliculorum rostralium (CCoR) (Figures 8C,D) as well as two pairs of brachia were also identifiable – the brachium colliculi rostralis (BCoR) only in MAC (Figure 8B) and brachium colliculi caudalis (BCoC) in both methods (Figures 8A–C, 9B).

Out of the tegmentum the substantia nigra (SN) is evident in both methods (Figures 7C,D, 8A,B,D). The nucleus ruber (NR) is the second most prominent structure depicted (Figures 7C,D, 8). The substantia grisea centralis (SGC) apparent in MAC was also identifiable in MRI as hypointense area (Figures 8, 9A,B) that surrounds AqC that forming a zone without signal (Figures 7C,D, 8, 9A,B). A very distinctive nucleus interpeduncularis (NIp) in MAC is also depicted in MRI, due to a characteristic hypointense rim (Figures 8B–D). The nucleus motorius nervi oculomotorii (NMNO) was identified only in MAC (Figures 7D, 8B,D). Area of the formatio reticularis mesencephali (FRM) was approximated (Figures 8B,D, 9A,B). CrC were easily identified due to specific topography and histology in MAC and as a highly hypointense areas in MRI (Figures 7, 8, 9A,B). The decussatio pedunculorum cerebellarium rostralium (DPCR) was also depicted in both methods (Figures 8C,D, 9B). The fasciculus longitudinalis medialis (FLM) (Figures 8D, 9B) was identifiable only in MAC images; on the other hand, lemniscus lateralis (LL) was detected by MRI only (Figure 9A).

### Cerebellum – Gross and Internal Anatomy

The hemisphaeria cerebelli (HC) are quite laterally expanded and the fissura prima (FPr) takes a U-shaped course (Figure 1A). The vermis (Vm) is noticeably divided into lobules (Figure 1E). Lobules from the caudal and dorsal aspects are listed from ventral to dorsal: a poorly separated uvula vermis (UVm), a quite well separated pyramis vermis (PVm), an apparently protruding tuber vermis (TVm), two lobuli poorly separated one from another – folium vermis (FVm) and declive (Dc) are eventually distinctly separated from the culmen (Cu) by the abovementioned FPr.

**FIGURE 10:**
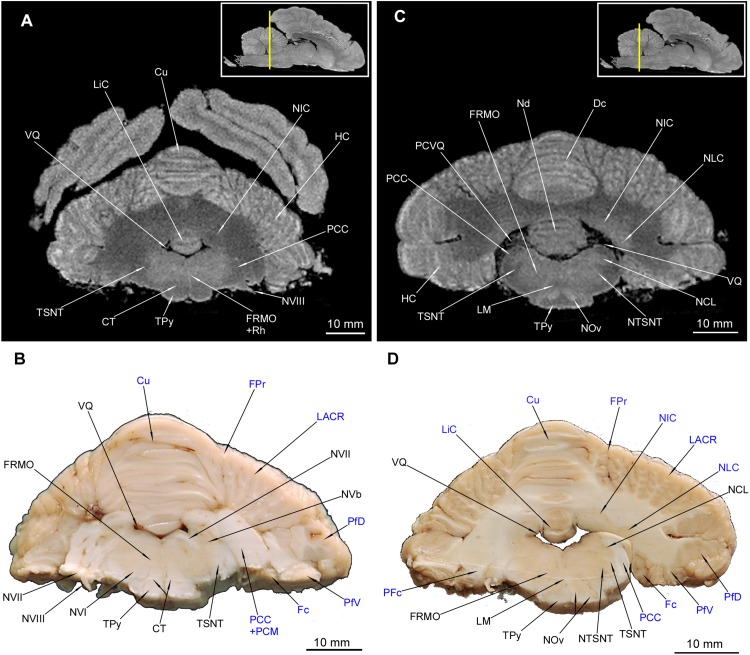
MRI imaging and cross sections through the middle part of cerebellum and rostral and middle parts of medulla oblongata in *U. arctos.* **(A,B)** MRI and macroscopic anatomy cross-section through the middle part of cerebellum and the most rostral part of medulla oblongata. The correspondence between **(A)** and **(B)** was made according to the medulla oblongata. The hemisphaeria cerebri are still visible in **(A)**. Note the well discernible corpus trapezoideum and slightly delineated by the signal difference nucleus interpositus cerebelli in **(A)**. Nearly all area of the medulla oblongata is hyperintense due to the formatio reticularis. **(C,D)** MRI and macroscopic anatomy cross-section through the middle part of cerebellum and rostral part of medulla oblongata. The correspondence was made according to the medulla oblongata, whereas the cerebellar cross-section is slightly offset so that it meets more the correspondence with **(A)**. Note the hyperintense nuclei olivares in **(C)** with the hilum **(D)** as well as very evident cerebellar nuclei in **(C,D)**. In **(D)** the right transition of pedunculus cerebellaris caudalis into white matter of cerebellum was captured. The insets in **(A,C)** shows the exact plane of cross-section in MRI and the best achieved correspondence in macroscopic anatomy **(B,D)**. In **(B,D)** the cerebellar structures were written in blue color. CT, corpus trapezoideum; Cu, culmen; Dc, declive; Fc, flocculus; FPr, fissura prima; FRMO, formatio reticularis medulla oblongatae; HC, hemisphaerium cerebelli; LACR, lobulus ansiformis, crus rostrale; LiC, lingula cerebelli; LM, lemniscus medialis; NCL, nucleus cuneatus lateralis; Nd, nodulus; NIC, nucleus interpositus cerebelli; NLC, nucleus lateralis cerebelli; NOv, nucleus olivaris; NTSNT, nucleus tractus spinalis n. trigemini; NVb, nuclei vestibulares; NVI, nervus abducens; NVII, nervus facialis; NVIII, nervus vestibulocochlearis; PCC, pedunculus cerebellaris caudalis; PCM, pedunculus cerebellaris medius; PCVQ, plexus chorioideus ventriculi quarti; PFc, pedunculus flocculi; PfD, paraflocculus dorsalis; PfV, paraflocculus ventralis; Rh, rhaphe; TPy, tractus pyramidalis; TSNT, tractus spinalis n. trigemini; VQ, ventriculus quartus.

**FIGURE 11:**
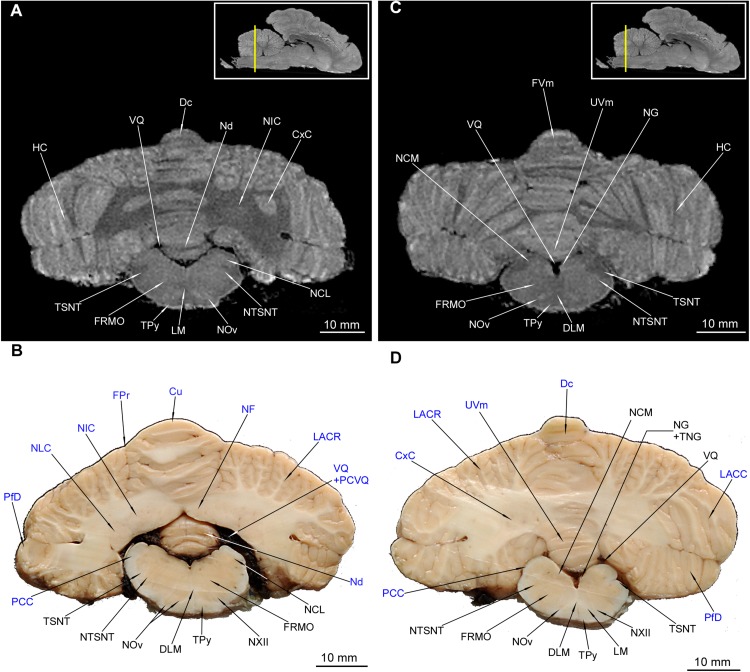
MRI imaging and cross sections through the middle and caudal parts of cerebellum and middle and caudal parts of medulla oblongata. **(A,B)** MRI and macroscopic anatomy cross-section through the middle parts of cerebellum and medulla oblongata. The correspondence between **(A)** and **(B)** was made according to the medulla oblongata. Note the extensive area of the cerebellar nuclei in **(B)**. In **(A)** hyperintense zones are the indentations of cerebellar cortex that are also visible in **(D)**. The nuclei olivaris is visible in its greatest expansion with its hilum discernible also in MRI **(A)**. Thin strips of the intramedullar fibers of nervus hypoglossus are visible in **(B)**. **(C)** and **(D)** MRI and macroscopic anatomy cross-section through the caudal parts of cerebellum and medulla oblongata. The correspondence between **(C)** and **(D)** was made according to the medulla oblongata. Note the transition of left tractus spinalis nervi trigemini from the inside the outside of the medulla oblongata **(D)**. The nucleus gracilis is very evident in MRI **(C)**. The cerebellar white matter in **(C)** is no longer visible whereas it is still captured in **(D)**. The insets in **(A,C)** shows the exact plane of cross-section in MRI and the best achieved correspondence in macroscopic anatomy **(B,D)**. In **(B,D)** the cerebellar structures were written in blue color. Cu, culmen; CxC, cortex cerebelli; Dc, declive; DLM, decussatio lemniscorum medialium; FRMO, formatio reticularis medulla oblongatae; FPr, fissura prima; FVm, folium vermis; HC, hemisphaerium cerebelli; LACC, lobulus ansiformis, crus caudale; LACR, lobulus ansiformis, crus rostrale; LM, lemniscus medialis; NCL, nucleus cuneatus lateralis; NCM, nucleus cuneatus medialis; Nd, nodulus; NF, nucleus fastigii; NG, nucleus gracilis; NIC, nucleus interpositus cerebelli; NLC, nucleus lateralis cerebelli; NOv, nucleus olivaris; NTSNT, nucleus tractus spinalis n. trigemini; NXII, nervus hypoglossus; PCC, pedunculus cerebellaris caudalis; PfD, paraflocculus dorsalis; PCVQ, plexus chorioideus ventriculi quarti; TNG, tuberculum nuclei gracilis; TPy, tractus pyramidalis; TSNT, tractus spinalis n. trigemini; UVm, uvula vermis; VQ, ventriculus quartus.

**FIGURE 12:**
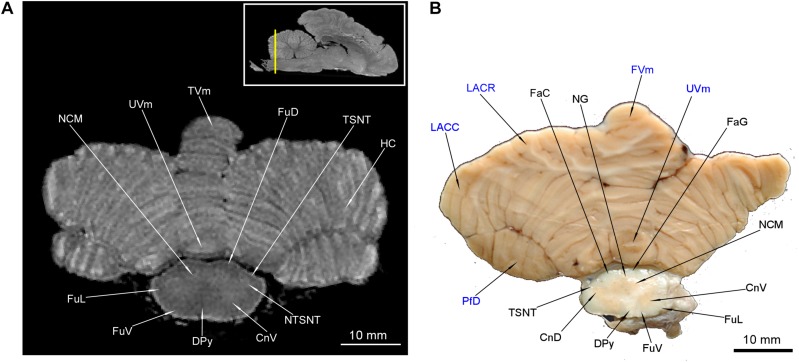
MRI imaging and cross sections through the most caudal part of cerebellum and the medulla oblongata – medulla spinalis transition. Note the decussatio pyramidum and gray matter typical for the medulla spinalis. The dorsal gray matter is typical for the nuclei of the medulla oblongata. The gray matter of cerebellum is no longer visible. In **(B)** the cerebellar structures were written in blue color. The inset in **(A)** shows the exact plane of cross-section in MRI and the best achieved correspondence in macroscopic anatomy **(B)**. CnD, cornu dorsale; CnV, cornu ventrale; DPy, decussatio pyramidum; FaC, fasciculus cuneatus; FaG, fasciculus gracilis; FuD, funiculus dorsalis; FuL, funiculus lateralis; FuV, funiculus ventralis; FVm, folium vermis; LACC, lobulus ansiformis, crus caudale; LACR, lobulus ansiformis, crus rostrale; NCM, nucleus cuneatus medialis; NG, nucleus gracilis; NTSNT, nucleus tractus spinalis n. trigemini; PfD, paraflocculus dorsalis; TSNT, tractus spinalis n. trigemini; TVm, tuber vermis; UVm, uvula vermis.

Parts of HC (Figures 1A,E) were also depicted: the most prominent lobulus ansiformis (LA) divided into crus rostralis (LACR) and crus caudalis (LACC). A medially to LACC located lobulus paramedianus (LPm) bordering laterally with UVm, PVm and TVm. On the ventral surface the paraflocculus dorsalis (PfD) and a more ventro-rostrally lying paraflocculus ventralis (PfV) were found (Figures 1D,E). The abovementioned parts of HC were also seen in MAC images (Figures 9D, 10C,D, 11C,D, 12B) whereas the lobuli of Vm are apparent in MAC and MRI (Figures 9**–**12A,B). Other parts of HC, visible only in MAC are the flocculus (Fc) with its pedunculus flocculi (PFc) (Figures 10B,D), the lingula cerebelli (LiC) (Figures 10A,D) and the nodulus (Nd) (Figures 10C, 11A,B).

The cerebellar nuclei were also detected. The nucleus fastigii (NF) was identifiable only in MAC (Figure 11B). Another two structures could be recognized with both methods – the nucleus interpositus cerebelli (NIC) (Figures 10A,C,D, 11A,B) and the nucleus lateralis cerebelli (NLC) (Figures 10A,C,D). It is worth to mention that in Figures 11A,D parts of cerebellar cortex were depicted as indentations into the white matter from the back – therefore they resemble the cerebellar nuclei. The pedunculi cerebellares were distinct: rostralis (PCR), medius (PCM) (Figures 9C,D) and caudalis (PCC) (Figures 10, 11B,D).

### Pons and Medulla Oblongata

The pons is visible on the ventral surface as distinctive bulge with transversely coursing fibers, including a medially coursing sulcus basiliaris (SBa) and the arteria basiliaris on its surface (Figure 1D). At the rostral margin of the pons, a portion of the nervus trigeminus (NV) was detected. A thin nerve visible on the one side is probably the nervus abducens (VI) (NVI). Two small bulges bordering with the caudal margin of the pons are the pyramides (Py) of the medulla oblongata (MO).

Only a few structures of the pons were identifiable. Within the pars ventralis pontis, we found the nuclei pontis (NPo) (Figures 8C,D, 9) and the fibrae pontis transverse FPoT) (Figures 8C,D, 9). For the pars dorsalis pontis, we could find the rhaphe (Rh) (Figures 9C,D), but the nuclei rhaphe (NRh) were visible only in MRI (Figure 9C). Some of gray matter within MO was also identifiable in cross-sections. The most prominent was the nucleus olivaris (NOv) (Figures 10C,D, 11) with its hilus (HNOv) apparent in MAC but also in one MRI image (Figure 11A). The nucleus cuneatus lateralis (NCL) forms a well identifiable gray matter over a long distance (Figures 10C,D, 11A,B). The nucleus cuneatus medialis (NCM) was also detected (Figures 11C,D, 12A,B). The nucleus gracilis (NG) was more apparent in MRI (Figures 11C,D, 12B) due to the bulge it forms on the fossa rhomboidea – tuberculum nuclei gracilis (TNG). A highly hyperintense asymmetrical area visible in Figure 9A is probably a lesion. The nuclei vestibulares (NVb) were collectively intentified only in MAC near the ventriculus quartus (VQ) (Figure 10B). TSNT along with the nucleus tractus spinalis nervi trigemini (NTSNT) were apparent in MAC and MRI, across the medulla oblongata and end segment of the pons (Figures 9C,D, 10–12).

The tractus pyramidalis (TPy) is evident in both methods (Figures 9–11) coursing through entire pons and MO. The corpus trapezoideum (CT) was also identified in both methods (Figures 10A,B). Intramedullar cranial nerve fibers were identifiable only in MAC: the nervus facialis (NVII) (Figure 10B), the nervus hypoglossus (NXII) (Figures 11B,D) and the nervus vestibulocochlearis (NVIII) (Figure 10B). The latter was partially obscured in MRI, we could detect it only on the ventro-lateral surface of MO (Figure 10A). The lemniscus medialis (LM) was depicted with the decussatio lemniscorum medialium (DLM) (Figures 10C,D, 11). In the most caudal part of MO the funiculi dorsales (FuD), ventrales (FuV) et laterales (FuL) were prominent in MAC and MRI (Figure 12). The division of FuD into the fasciculus gracilis (FaG) and fasciculus cuneatus (FaC) was observed in MAC (Figure 12B).

Area of formatio reticularis pontis (FRPo) et medullae oblongatae (FRMO) was also detected and is mainly identifiable in MRI as a weak contrast area (Figures 9C,D, 10, 11). VQ (Figures 10, 11) contains the plexus chorioideus ventriculi quarti (PCVQ) (Figures 10C, 11B,D). The velum medullare rostrale (VMR) was only identified in MAC.

## Discussion

### Telencephalon

#### The Brain Surface

Sulci and gyri of the telencephalon were depicted only in macroscopic anatomical cross-sections (MAC) but all of them are also distinct in MRI cross-sections, showing good correspondence. The brain surface of the brown bear is highly visually convoluted with a presence of secondary sulci and gyri. It is more convoluted with respect to the dog (Uemura, 2015), the cat (Smith et al., 2001) or to Mustelidae (Radinsky, 1975) and Viverridae species (Radinsky, 1975). However, it is less convoluted than in Pinnipedia: *Zalophus californianus* (Montie et al., 2009), northern fur seal (*Callorhinus ursinus*) and Steller sea lion (*Eumetopias jubatus*) (based on specimens available in CMBC). Gyrification studies showed the polar bear (*Ursus maritimus*) having a considerably more convoluted brain surface than other sampled Carnivora species, surpassed only by two Pinnipedia species (Lyras et al., 2016).

The lateral surface of the hemisphere (Figure 1B) shows a smaller number of gyri than that in the dog (Uemura, 2015). FP in *U. arctos* is outflanked immediately by GEsR and GEsC. The gyrus sylvius rostralis et caudalis along with sulcus ectosylvius rostralis et caudalis that respectively border GEsR and GEsC laterally in the dog are absent in *U. arctos.* The same observation was made for giant panda (*Ailuropoda melanoleuca*) (Mettler and Goss, 1946; Dong, 2008), *U. maritimus* (Dong, 2008) and the sun bear (*Helarctos malayanus*) (Kamiya and Pirlot, 1988b). The consequence of that reduction is lowering of SSsR and SSsC from dorsal to lateral surface of the hemisphere, their considerable shortening and shape change in comparison to the dog (Uemura, 2015) and the cat (Smith et al., 2001). SSsR and SSsC are together arcuate in the cat and dog, but in *U. arctos* are more hairpin-shaped, therefore the sulcus suprasylvius medius is absent. The same is true for other Ursidae specimens available in CMBC and as shown in other studies (see Mettler and Goss, 1946; Dong, 2008). Lateral areas of brain in *H. malayanus* as well as four other species making up the ursiformes group [*A. melanoleuca*, the American black bear *Ursus americanus*, the red panda *Ailurus fulgens* (Ailuridae) and the racon, *Procyon lotor* (Procyonidae)] have been investigated (Kamiya and Pirlot, 1988b). The shape of fissura pseudosylvia and adjacent gyri were compared, and brain regions with putative somatosensory and motor area (gyrus suprasylvius rostralis = gyrus coronalis) appeared more expanded than in Canidae, assigned to ursiformes’ more skillful use of hands and fingers.

Similar low-lying course, shape and shortening are present in many Mustelidae species including *Gulo gulo*, *Taxidea taxus*, *Meles meles*, and *Arctonyx collaris* (Radinsky, 1973a). The lack of medial structure of the gyri ectosylvius et suprasylvius is evident in *U. arctos* in comparison to the dog (Uemura, 2015). The shape of FP varies within genera, as noted by Kamiya and Pirlot (1988b). In *U. arctos*, like *U. americanus*, the FP is tighter at full length, whereas in *A. melanoleuca* for the ventral 2/3 of its length it is slightly gapped, between GEsR and GEsC. FP in *H. malayanus*, *A. fulgens* and *P. lotor* is slightly open for nearly its full length. SC as a rostral continuity of SM, starting from SA convergence (Uemura, 2015), is marked by a short medial branch in the middle of the course (Figure 1B). The branch is also present in other Ursidae – notably, in *A. melanoleuca* it was exceptionally prominent, splitting the gyrus sigmoideus into two parts (Mettler and Goss, 1946). The branch is less prominent in *H. malayanus* (Kamiya and Pirlot, 1988b) but still stronger than in *U. arctos, U. maritimus* and *U. americanus* in CMBC.

A signature trait of the Ursidae and Pinnipedia brain is a pronounced development of the gyri frontales (Figures 1A,C). Mettler and Goss (1946) marked the gyri with terms “superior,” “medius” and “inferior”; notably, the gyrus frontalis is not included in NAV. We used the terms “dorsalis” and “ventralis” in lieu of “superior” and “inferior” to unify the nomenclature. The left and right GFD together form the signature “ursine lozenge” as termed by Mettler and Goss (1946). The structure, GFM and GFV are all apparent in Ursidae (Mettler and Goss, 1946; Kamiya and Pirlot, 1988b; Welker et al., 2009) and Pinnipedia (Montie et al., 2009; Welker et al., 2009). On the other hand only one gyrus frontalis is present in the dog (Meyer, 1964; Uemura, 2015) and in the cat none of the gyri frontales is observed (Smith et al., 2001; Kobryń and Kobryńczuk, 2004). The course of SCr (Figure 1A) is variable in our samples. SCr is defined as deep sulcus running from the medial surface of the hemisphere laterally (Meyer, 1964). In our specimen (RH0178-15) the marked SCr begins bilaterally in medial surface of the hemispheres, similar to *U. maritimus* in CMBC and *H. malayanus* (Kamiya and Pirlot, 1988b). The situation is complicated by somewhat wedged GFD into SCr (Figures 1B,C). In RH0178-15, RH0156/13, and in *U. maritimus* in CMBC the rostral sulcus to GFD begins in the medial surface of the hemispheres. In the same *U. maritimus* the caudal sulcus to GFD merge with the SPc and the same state we have observed on either side of the RH0178-15 brain (Figure 1A). However, in another specimen of *U. arctos* (RH194/14) the caudal sulcus to GFD is apparent at the medial surface. Dong (2008) also depicted the caudal sulcus as SCr in *A. melanoleuca* (2008), while Mettler and Goss (1946) depicted only the lateral section of SCr, suggesting the problematic medial section was omitted from their study. We suggest three possible models: (a) rostral sulcus to GFD is the homolog of SCr; (b) caudal sulcus to GFD is the homolog; (c) both caudal and rostral sulci to GFD are the homolog of SCr present in the other Carnivora that was separated by the developing GFD. The issue can be elucidated by further cytoarchitectonic or gyrification studies in *U. arctos.* Interestingly, the rostral area to SCr is supposedly involved in social behavior, as studied in *Crocuta crocuta* (Holekamp et al., 2007; Sakai et al., 2011) and in *Panthera leo* (Sakai et al., 2016). The area would be substantially larger if GFD is also included.

Another transverse sulcus – SCrM, is defined in the dog as the sulcus beginning from about the middle of SCi and reaching the dorsal margin of the brain at the level SA (Meyer, 1964). SCrM is well developed in the examined *U. arctos* specimen (Figure 1A) as well as in *A. melanoleuca* (Mettler and Goss, 1946), *U. maritimus* available in CMBC and *H. malayanus* (Kamiya and Pirlot, 1988b). It is plausible that SCrM is exceptionally well developed laterally in Ursidae as in all abovementioned species it reaches the dorsal surface of the brain and separates the caudally lying GM from the rostrally lying GPc (Figure 1A). SCrM is absent in the cat (Smith et al., 2001; Mogicato et al., 2011b; Gray-Edwards et al., 2014), Viverridae (Radinsky, 1975), and Mustelidae (Radinsky, 1973a). Pinnipedia species’ SCrM is apparent, suggesting the presence of the sulcus is typical of Caniformia. We did not observe dimples in the anterior section of *U. arctos* telencephalon, although they are apparent in Mustelidae (Radinsky, 1973a) and Hyaenidae (Holekamp et al., 2007; Sakai et al., 2011). SPs in *U. arctos* is relatively short (Figures 1B,C) in comparison to that in the ferret (*Mustela putorius furo*) (Sawada et al., 2013), other Mustelidae including *T. taxus*, *M. meles* (Radinsky, 1973a) as well as numerous Viverridae including *Hemigalus derbyanus*, *Diplogale hosei* and Eupleridae, e.g., *Fossa fossa* (Radinsky, 1975). SPs is exceptionally long and arcuate reaching up to the dorsal surface of the hemisphere. GM in *U. arctos* (Figure 1A) is more elaborate than that in the dog (Whalen, 2003; Uemura, 2015) because of its secondary division into two unequal parts. Some Ursidae show a longitudinal discontinuity, e.g., CMBC brains of *H. maritimus* and *U. americanus* and *A. melanoleuca* (Mettler and Goss, 1946) supporting the notion that the gyrus assumes a sigmoid course in many carnivores. The dog GM appears divided into nearly equal parts by the sulcus endomarginalis (Meyer, 1964). The rostral continuity of GM is GSC (Meyer, 1964), however the term “gyrus sigmoideus” is not included in NAV. According to Whalen (2003) in the online atlas a whole gyrus caudal to SCr is termed as the “GPc” and rostral to SCr as the “gyrus praecruciatus”. Meyer (1964) defined GPc as an additional gyrus, located caudally to SPc if the sulcus is present. We retained the term “GSR et caudalis” for the rostral and caudal gyri to the SPc and “GPc” for the gyrus immediately caudal to SPc because we observed this anatomical trait consistently across Ursidae.

The caudal part of the cerebral hemisphere is the most problematic. We depicted GOc following the online atlas of Whalen (2003) considering it as continuity of GM onto the caudal surface of the hemisphere (Figures 1A,E). Smith et al. (2001) used the same term in their description of the cat brain surface. An alternative term might be the “gyrus postmarginalis” (in former papers “gyrus postlateralis”) (Meyer, 1964). However, the sulcus separating GM from GEm in *U. arctos* does not constitute a natural continuity of SM, thus we prefer the terms GOc and SOc, denoting the lateral course of the SOc. The evident narrowing separating GM from GOc supports our thesis – in the studied specimen the narrowing is visible better on the left side (Figures 1A,E). Another doubtful gyrus is GEm- depicted by us after Mettler and Goss (1946) and Dong (2008) (Figure 1B). If the gyrus were to be a homolog of that in the dog (Uemura, 2015) only its caudal part would be present in *U. arctos.* Instead, both GM and GEm seem to constitute a single complex gyrus lacking a strict border between them. In other Ursidae the situation is also complicated (Mettler and Goss, 1946; Kamiya and Pirlot, 1988b). To fully resolve the issue of posterior gyri homologies, further cytoarchitecture studies are required.

#### Rhinencephalon

BO (Figures 1B,D, 2A,C,D) was shown only in single MAC due to technical difficulties. It is comparatively apparent in both types of cross-sections. Portions of BO were not preserved, precluding inference of the relative volume of the structure in other Carnivora. In general, the BO in Ursidae is relatively large (Mettler and Goss, 1946; Kamiya and Pirlot, 1988b) and its caudal region is cylindrical, as found in the dog (Whalen, 2003; Uemura, 2015), *H. malayanus* (Kamiya and Pirlot, 1988b) and *U. americanus* (Welker et al., 2009). A relatively large BO with relatively short PO is present in many lesser, macrosmatic Carnivora representatives – Mustelidae (Radinsky, 1973a), Viverridae (Radinsky, 1975) or Felidae available in CMBC. BO in *A. melanoleuca* is more spherical and rostrally centralized with a very prominent PO containing a distinct narrowing (Mettler and Goss, 1946). On the other hand, BO in Pinnipedia is relatively smaller, reflecting the decrement in olfaction importance in that group (Montie et al., 2009). PO is easily distinguishable in MAC (Figure 3B), but in MRI it is barely separated from the brain surface, similar to telencephalic gyri. TOL and TOI are both apparent in both MAC and MRI, due to their hypointensity (Figures 3C,D, 4, 5). Initial sections of TOI are more evident in MRI and terminal sections of TOL were distinct only in MAC (Figures 5B,D). TOI poses a hallmark for macrosmatic Carnivora (Kobryń and Kobryńczuk, 2004). TOM was found only in MAC (Figure 4D); TO is visible in both methods (Figures 4D, 5) but the MRI signal interfusion with NC is possible (Figure 5A). TO in *U. arctos* is nearly perfectly round and that shape seems to be feature of Ursidae – the same is observed in *A. melanoleuca* (Mettler and Goss, 1946) and *H. malayanus* (Kamiya and Pirlot, 1988b). LP assumes the fist-like shape in *U. arctos* (Figure 1D) as well as in other Ursidae (Mettler and Goss, 1946; Kamiya and Pirlot, 1988b; Welker et al., 2009). Its longitudinal measure is relatively shorter than that in *C. lupus*, *Vulpes zerda*, *Canis latrans* available in CMBC, *Proteles cristata* (Flower, 1869) and *A. fulgens* (Flower, 1870). LP seems to be smaller in relation to the whole brain in *U. arctos* than in abovementioned Canidae species as well as in Mustelidae (Radinsky, 1973a) and Viverridae (Radinsky, 1975). The LP is relatively larger than in Pinnipedia, although it shares a similar shape. Further volumetric analysis of *U. arctos* brain would provide information about relative volume of rhinencephalon and, consequently, about comparative importance of olfaction in Ursidae. Dong (2008) studied brains of panda ancestors, †*A. baconi* and †*A. microta* (both extinct), as well as giant panda and polar bear using virtual endocasts (endocranial casts acquired with CT). This volumetric analysis proved that the polar bear has the largest rhinencephalon among the abovementioned ursid species.

#### Subcortical Gray Matter

Accurate borders of Pu are possible to delineate only in MAC (Figures 4B,D, 5B,D, 6B,D, 7B). It is also apparent in MRI, usually along with GP (Figures 4A,C, 5A,C, 6C) however, its borders are less clear, see Figure 5C. Both structures form a hyperintense zone in medio-ventral part of the telencephalon. It was possible to distinguish Pu from NC (converging in the initial section) in MAC (Figure 4B) whereas in MRI both structures form one fused hyperintense zone – we labeled the zone as Pt + NC (Figure 4A). Pu in *U. arctos* always lies ventro-medially to Cm and assumes shapes from lentoid to triangular. Pu was also detected in the dog (Mogicato et al., 2011b) using unstained cross-sections and is discernible in MRI, in the cat (Gray-Edwards et al., 2014) and in *Z. californianus* (Montie et al., 2009). However, in the dog it was either undetected (Mogicato et al., 2011b) or collectively depicted as the nucleus lentiformis (Leigh et al., 2008). Borders of GP (Figures 4C,D, 5, 6) are also vague in MAC but it forms a consistently lighter area than Pu, in medial proximity. After staining, improved contrast between gray and white matter it is easily distinguishable (Mogicato et al., 2011b). GP in MRI is often discernible as the nucleus lentiformis but in the California sea lion, *Z. californianus*, it was possible to detect both the structures (Montie et al., 2009). We also managed to discern Pu and GP separately in one of MRI section (Figure 5C). Cm is lying under the sulcus rhinalis lateralis (that is the pallium dorsale – pallium laterale interface) and as a distinct structure, more evident in MAC (Figures 4B,D, 5B,D, 6B,D, 7B,D) with an apparent CEx laterally and capsula externa medially to it. Cm in MRI usually resemble the cerebral cortex thickening (Figures 5C, 6A,C, 7A). Cm signal intensity may be slightly lower than cortex intensity – then it is a little more difficult to spot but it does not form a deceptive cortex thickening (Figures 5C, 6A). It is extremely difficult to distinguish CEx (Figure 5A) and CE (Figure 6A) as hypointense narrow strips. Cm in its initial section is crescent-shaped in *U. arctos* (Figures 4B,D, 5B,D, 6B,D), whereas more lentoid in its caudal parts (Figures 7B,D). Cm was also visualized in the dog (Fletcher, 2007; Mogicato et al., 2011a) and in the cat (Smith et al., 2001; Gray-Edwards et al., 2014) and the vertical measure of Cm in both these species is greater than in *U. arctos*, more pickaxe-shaped than crescent-shaped. In other MRI studies, Cm is poorly discernible (Leigh et al., 2008; Kang et al., 2009; Mogicato et al., 2011b) or undetectable (Mogicato et al., 2011a). Anterior and middle parts of the NC (caput et cauda) are very well identifiable in both methods (Figures 4, 5, 6A,B,D) and in MRI are hyperintense. The signal appears faded for the cauda nuclei caudati and is more difficult to observe in MRI (Figures 6C, 7A). In MAC it is evident near the lateral ventricle wall but evinces lesser diameter (Figures 7B,D). The caput of NC occupies a broad periventricular area and assumes a somewhat triangular shape. The area decreases for the corpus of NC and the cauda of NC forms merely a thin periventricular strip of gray matter. NC was also visible in unstained cross-sections and MRI in the cat (Gray-Edwards et al., 2014) and the dog (Mogicato et al., 2011b) as well as in MRI of *Z. californianus* (Montie et al., 2009) and the ferret (Sawada et al., 2013). Only Mogicato et al. (2011b) have not visualized NC in MRI, but hardly any other subcortical telencephalic structure was apparent. The shape and relative volume of NC, in proportion to the whole telencephalon, is very similar between *U. arctos*, the dog (Mogicato et al., 2011b), the cat (Gray-Edwards et al., 2014) and the ferret (Sawada et al., 2013). The comparison of NC in Ursidae and the ferret is stymied – Sawada et al. (2013) measured NC and nucleus lentiformis volume to the whole encephalon, whereas Kamiya and Pirlot (1988b) measured the striatum proportion in *A. melanoleuca*, *U. americanus H. malayanus* without clarification of the term “striatum.” The striatum may involve other structure in surrounding tissues, depending on interpretation (Butler and Hodos, 2005).

NAc is usually defined as separate structure within NC, in the form of a thin strip within medial and lateral wall of the VL (Kobryń and Kobryńczuk, 2004). A noteworthy identification of NAc in MRI, where its borders are sharp and signal is strong (Figure 5C). In MAC its borders are unclear (Figure 5D) and distinction between NAc and ventral part of NC is difficult. NAc in *U. arctos* extends in both walls of VL near TOI and appears congruent to the structure depicted by Fletcher (2007) in the dog. CAg was observed in both methods but is more evident in MAC, where its nuclei with interwoven strips of white matter are visible (Figures 6B,D). The mosaic is not observed in MRI but in general, the signal of CAg is quite strong within the LP rostro-laterally to the incipient hippocampal formation (Figures 5C, 6A). CAg was also observed in MRI in the dog (Leigh et al., 2008), the cat (Gray-Edwards et al., 2014), *Z. californianus* (Montie et al., 2009) and the ferret (Sawada et al., 2013) with similar localization, extent and general appearance. SeC is easily identified in both methods (Figures 4D, 5). SeP was absent – none of the traced MRI cross-sections contained the structure nor the other examined specimens with macroscopic anatomy method (RH0156/13, RH155/14, RH194/14). It is plausible that SeP is then highly reduced in *U. arctos* in a manner that CC is directly adjacent to SeC and Fx. However SeP is present in *U. maritimus* seen in CMBC and easily discernible in three unidentified cross-sections. Also it is present and prominent in *Z. californianus* (Montie et al., 2009) and appears relatively short in the dog (Meyer, 1964; Fletcher, 2007). Its presence was not mentioned in papers devoted to the cat (Mogicato et al., 2011a; Gray-Edwards et al., 2014) and we investigated CMBC (Welker et al., 2009) to corroborate that indeed it is also absent in the cat.

*Hippocampal formation* is easily identifiable in MRI due to alternate array of hypo- and hyperintense strips (Figures 6C, 7A,C, 8A). Cortical parts of hippocampus are hyperintense with the gyrus dentatus (GD) evincing the least intensity of them and therefore the GD is more difficult to spot (Figures 7A,C). The alveus hippocampi (AH) is also difficult to descry. GD on the other hand is very apparent in MAC, as are AH, cornu ammonis (CA) and subiculum (Sb) (Figures 6D, 7B,D, 8B). The dorsal part of the hippocampal formation, however, scant, is easy to identify in MAC. In MRI the dorsal part is hypointense, with a barely discernible hyperintense strips corresponding to cortical parts but the more accurate identification is impossible due to weak contrast (Figure 7C). The tissue of the hippocampal formation evinces localization typical for Carnivora – both ventral and dorsal, which stems from the curvature of the structures along Fx. In fact, the cross-sections involve the same structures captured in two locations and referred to as the dorsal and ventral hippocampus (Butler and Hodos, 2005). The ventral hippocampus (VH) is more predominant in *U. arctos* than in the dog (Uemura, 2015), the cat (Gray-Edwards et al., 2014), *T. taxus* (Welker et al., 2009) and the ferret (Sawada et al., 2013) where the dorsal (DH) and VH are comparably well developed. On the other hand, in *Z. californianus* nearly all hippocampal tissue is localized ventrally with a scant amount dorsally (Montie et al., 2009). DH in *U. arctos* containing gray matter is localized largely caudally (Figures 7D, 8B). It distinguishes its organization from that in the dog, cat and Mustelidae and situate closer to Pinnipedia. Reduction of DH in *U. arctos* is not that advanced than in Pinnepedia.

#### White Matter

CC due to obvious topography is easily identified in both methods (Figures 4–7, 8B), being hypointense in MRI, like all the white matter of the hemispheres. However, in MAC, transverse course of fibers is apparent because of sharpening of the borders between the corpus callosum and the remaining white matter. In Figure 4 the genu, a knee-like structure of CC, was captured. The only difference is the vicinity of Fc in *U. arctos* with absent SeP unlike in most other Carnivora. All parts of Fx were depicted (Figures 6, 7). The signal intensity of Fx is comparable with CC, but a distinctive narrowing facilitates delineating the border between these two structures (Figure 6A). Dorsal part of Fx is comparatively well discernible in both methods (Figures 6A,B), but ventral parts due to hypointensity are better discernible in MRI (Figure 6A). The crura fornicis (CrFx) are laterally expanded, however, as mentioned above, the hippocampal tissue is not present until the most caudal sections of Fx with its caudal part associated with hippocampal tissue is more evident in MAC (Figure 7D). In MRI they are hypointense, similar to the gray matter of DH, resulting in less clear borders. The CEx is generally difficult to observe in MRI (this study). It was undetectable in the cat (Gray-Edwards et al., 2014) and in the dog (Leigh et al., 2008). The same goes for CE (Figures 4D, 5C,D, 6A,B,D, 7B). The most prominent is CI (Figures 4–6, 7A,B,D) and in Figures 7B,D its transitions into the left and right CrC were captured. Unlike the other capsules, CI is apparent in MRI (Leigh et al., 2008).

### Diencephalon

#### Thalamus and Subthalamus

Rostral parts of Th are more evident in MAC where the border between Th and a surrounding CI is easier to delineate (Figure 6D). Caudal structures are belied by non-homogeneous texture stemming from the presence of multiple thalamic nuclei groups (Butler and Hodos, 2005). The borders between nuclei groups are unclear, so we did not make an attempt to detect them without staining. Even stained tissue lacked definite thalamic nuclei (Fletcher, 2007; Mogicato et al., 2011a). Penetrating blood vessels are apparent in MAC as black dots (Figures 6D, 7B,D). Th in MRI is discernible as a broad central hyperintense area. The signal intensity is the highest in more medial parts and it gradually decreases laterally finally reaching CI hypointensity (Figures 6C, 7A). The intensity of Pv is lesser than the remaining parts of Th (Figures 7A,C). The adhaesio interthalamica (ATh) is vast in *U. arctos* (Figures 6C,D, 7A,B) and it considerably reduces the lumen of the VT. ATh is also broad in the dog (Fletcher, 2007), the cat (Gray-Edwards et al., 2014), *Z. californianus* (Montie et al., 2009), and *C. crocuta* (Welker et al., 2009). CSu was identified only in MAC as a separated area below Th and over the CI – CrC transition (Figure 7B). The label is consistent with that done by Fletcher (2007) and with the literature definition (Kobryń and Kobryńczuk, 2004). NEp is remarkably discernible in MRI as a hyperintense, oval zone medially to Pu – GP and dorsally to TOp (Figures 6A,C, 7A). NEp in MAC is also apparent, especially in its caudal segment (Figures 6B,D, 7B). NEp is the homolog of the lamina medullaris medialis of GP in Primates (Butler and Hodos, 2005). NEp was also depicted in the cat by Gray-Edwards et al. (2014) and in the dog (Fletcher, 2007). The zona incerta was not identified, suggesting why the amount of information about the other Carnivora subthalamus is scant.

#### Hypothalamus

Collectively, NHs are evident in both methods (Figure 6). No hypothalamic nuclei could be detected and depicted. Previously, it proved impossible to detect white and gray matter by contrast staining in the dog (Fletcher, 2007; Mogicato et al., 2011b). NHs are hyperintense in MRI and well contrasted with white matter in MAC. The hypophysis was not preserved during preparation and only If is depicted (Figure 1D). The latter was not captured in anatomical cross-sections (the cuts run exactly just before the rostral and caudal border). TC is prominent and features darker coloration in MAC than NH (Figure 6D). In MRI, the signal intensity is comparable (Figure 6C). CMas were cut in their caudal parts in a way that their gray matter is no longer visible in MAC (Figure 7B). CMas in MRI are apparent as paired highly hyperintense areas thus nuclei mamillares were probably involved (Figure 7A). Despite of the method CMa are easily identified due to paired bulges formed in ventral diencephalon. The feature typical for Carnivora (and a few other orders) is parity of CMa, as documented in the dog (Fletcher, 2007), the cat (Gray-Edwards et al., 2014), and *Z. californianus* (Montie et al., 2009). COp is discernible in MAC due to intensive white color highly contrasted with the surrounding tissues (Figures 5D, 6B) and in MRI it forms a round hypointense zone isolated ventrally from the rest of the brain (Figure 6A). TOp is more evident in MAC as it is highly contrasted and the border between it and ventral surface of the brain is sharp (Figures 6D, 7B,D). The contrast is poor in MRI and the border is more difficult to spot (Figures 6C, 7A,C).

#### Corpora Geniculata

The gray matter of CGL is featured by weak signal intensity in MRI (Figure 7C), whereas in MAC the contrast is very high, making identification of the pars dorsalis and pars ventralis nuclei geniculati lateralis (NGL) easier (Figure 7D). Similarly, TOp flanking lateral CGL was depicted in MAC, but showed little contrast in MRI. The nucleus geniculatus medialis (NGM) is also poorly discernible in MRI (Figure 7C) and in MAC the contrast is lower than that of NGL, however, it forms a lateral bulge. CGM that facilitates to indicate the structure accurately and it is apparent in the right side of Figures 7D, 8B and bilaterally in MRI (Figures 7C, 8A). LGN of *U. arctos* is similar in its relative volume to that in the dog (Whalen, 2003). The cytoarchitecture was indiscernible without staining but distinct division into its dorsal and ventral parts is evident. The comparison with LGN of other Carnivora (Sanderson, 1974) requires staining. Topographic relation of both CGL and CGM is similar in the dog – CGL forms an ovate bulge in the caudal diencephalon and CGM is collocated with the medially lying CoR (Whalen, 2003). All the structures are also discernible in MRI in the dog (Leigh et al., 2008) and the cat (Gray-Edwards et al., 2014) but only NGM is apparent in *Z. californianus* (Montie et al., 2009).

#### Epithalamus

GlPn was depicted only in MRI (Figure 7C). It forms a hyperintense round zone of considerable size, separated from the dorsal surface of the brainstem (Figure 7C). However, in *Z. californianus* the gland was even larger, occupying a broad area above CCa (Montie et al., 2009). Large volume of GlPn is a typical trait for Pinnipedia (Jasiński, 1987). Ha was depicted in both methods and appear hyperintense in MRI (Figure 7A). In MAC the contrast between Ha and Th is low but due to characteristic bulges, Ha is easily identified, regardless of the presence of its nucleus (NHa) (Figure 7B). NHa is discernible in MRI due to hyperintensity. CCa is apparent in both methods as a transverse hypointense in MRI band over VT – AqC transition (Figures 7C,D).

### Mesencephalon

#### Tectum

Out of tectal structures two pairs of colliculi were depicted. CoR evinces striatal organization visible in MRI as horizontally alternating hypo- and hyperintense bands (Figures 8A,C). The strata were also clearly visible to the naked eye in macroscopic investigation, although difficult to resolve in Figures 8B,D. CoC with NCoC takes a more ventro-lateral position to the former and are highly hyperintense in MRI (Figure 9A) and well discernible in MAC –captured in two cross sections (Figures 8D, 9B) – whereas NCoC is discernible only in Figure 9B. It is noteworthy that despite the hyperintensity in MRI, NCoC is only slightly contrasted with surrounding tissues in MAC, although its border is very sharp. Both CoR and CoC were also visualized in MRI in the cat (Gray-Edwards et al., 2014), the dog (Leigh et al., 2008), and *Z. californianus* (Montie et al., 2009). The size of both pairs of colliculi is similar in *U. arctos* and that is also observed in *Z. californianus* (Montie et al., 2009). However, in the dog, CoR is considerably less than CoC (Kobryń and Kobryńczuk, 2004). CCoR and CCoC are hypointense in MRI (Figures 8C, 9A) and in MAC they form typical transverse and well-discerned strips of white matter (Figures 8D, 9B). BCoR were apparent only in MAC and BCoC in both methods. The latter is identifiable in MRI because of the bulge it forms on the brainstem surface (Figure 8C). In MAC both pairs of brachia are apparent, including parts located within the mesencephalon (Figures 8B, 9B). Only CCoR and CCoC were discernible in MRI in the cat (Gray-Edwards et al., 2014). In other reviewed papers with MRI method white matter of the tectum was not depicted.

#### Tegmentum

Out of the tegmentum the most prominent structures were SN and NR. SN is very distinctive in MRI forming a hyperintense band dorsal to a highly hypointense CrC (Figures 7C, 8A). In MAC it also displays considerable contrast with surrounding white matter (Figures 7D, 8B,D). The area occupied by SN is expanded in Figure 7D where one can descry its pars reticulata ventrally and pars compacta dorsally. NR is not apparent in its initial sections in MRI (Figure 7C), although it forms a hyperintense zone dorsally to SN in further sections (Figures 8A,C). It is discernible in MAC as it forms relatively well-contrasted round zone with sharp boundaries (Figures 7D, 8B,D). In *U. arctos* NR assumes a beanlike shape. Inference of the shape of NR in other Carnivora is stymied because of difficulties with imaging it accurate borders in MRI, e.g., in the dog (Leigh et al., 2008) or lack of visualizing the structure at all, despite beautiful preparations performed by Gray-Edwards et al. (2014) in the cat. Stained cross-sections of other Carnivora species available in CMBS do not improve the ability to infer the NR borders, but the structure is generally discernible (Welker et al., 2009). In Figure 7D it is plausible that only left NR is captured whereas the right structure may be CSu because of slight section asymmetry and their relative vicinity (Kobryń and Kobryńczuk, 2004). Around AqC a ring of gray matter, SGC, was depicted. Due to its characteristic topography and cordiform shape it is easy to notice in both methods. SGC proved to be hypointense in MRI and its outer borders are nearly impossible to delineate (Figures 8A,C, 9A) in contrast to well-defined borders and high general contrast in MAC (Figures 7D, 8B,D, 9B). NIp assumes dark coloration and is very distinct from CrC in MAC (Figures 8B,D). NIp is also discernible in MRI, even though the signal intensity is comparable with that of FRM; a signature hypointense rim makes it possible to unambiguously localize the structure (Figure 8C). The IN is strongly stained in the dog where it takes a rectangular shape (Mogicato et al., 2011a), however in MRI in the *Z. californianus* (Montie et al., 2009), and the cat (Gray-Edwards et al., 2014) IN was not observed at all. The area typical for NMNO was detected (Figures 7D, 8B,D) and it was not possible to discern the structure in MRI. In Figure 7D NMNO forms an apparent wedged zone right below AqC and in further cross-sections it forms a less apparent zone ventrally to SGC. CrC due to its localization and uniform histology are easily identifiable in both types of cross-sections. They form hypointense semilunar or ovate zone in the ventral part of the mesencephalon (Figures 7A,C, 8A,C, 9A). Detection was more difficult in MAC, in more caudal sections with pars ventralis pontis inception – because they take up a more internal location (Figure 9B). A much smaller bundle – FLM, was discernible only in MAC as a subtle area of white matter just under SGC (Figures 8D, 9B). DPCR was detected in both methods. In MRI it appears to be a hypointense central zone with poorly defined boundaries (Figure 8C). In contrast, DPCR seems to form a broad light-colored central zone in MAC in the ventral part of the mesencephalon and dorsally to IN (Figures 8D, 9B). LL was identified only in MRI (Figure 9A) as a hypointense lateral band with relatively poor defined borders, as previously noted in MRI for *Z. californianus* (Montie et al., 2009) and the cat (Gray-Edwards et al., 2014) as one of very few structures better discernible in MRI than in unstained cross-sections.

### Pons

#### Pars Ventralis Pontis

Relatively few pontine structures were depicted, but all of them are discernible in both methods. Out of the pars ventralis pontis NPo occupy relatively large area. In MRI they form horizontal, hyperintense bands in ventral part of the brainstem (Figures 8C, 9A,C) and are quite well contrasted with surrounding white matter in MAC (Figures 8D, 9B,D). In Figures 8C,D, 9A,B two layers of nuclei (ventral and dorsal) are apparent with intervening FPoT. The abovementioned structures are collocated with CrC and their transition into TPy is visible in Figures 9A,D.

#### Pars Dorsalis Pontis

None of nuclei were depicted in *U. arctos* nor in any other species reported in available literature. However, hypointense congregation of white matter is discernible in MRI as PCM, PCR and TSNT. PCM forms a broad band of white matter within the lateral aspect of the brainstem that can be identified in MRI (Figure 9C). The transition between FPoT into PCM was recognizable in MAC (Figure 9D).

### Cerebellum

#### External Anatomy

The general shape of the cerebellum in *U. arctos* (Figures 1A,B,D,E) is conserved in other Ursidae – *U. maritimus, U. americanus* (Welker et al., 2009), *A. melanoleuca* (Mettler and Goss, 1946), and *H. malayanus* (Kamiya and Pirlot, 1988b). *U. arctos* differs from the dog in several anatomical features. The shorter relative length of the cerebellum relative to the decussatio pyramidum (DPy) lies below posterior part of the cerebellum (Figure 12), whereas in the dog it lies more caudally to its posterior ridge and the structures are never captured together in a cross-section perpendicular to the long axis of the medulla oblongata. HC are more expanded in *U. arctos* than in Mustelidae, including *M. nivalis, Neovison vison, T. taxus* (Welker et al., 2009) but considerably lesser than in Pinnipeds, possibly stemming from their aquatic lifestyle. The fraction of the cerebellum occupied by the cerebral hemispheres varies: in *U. arctos* about 1/3 volume, similar to that observed in other Ursidae and some Mustelidae (the ferret), and Pantherinae subflamily (*P. leo, P. pardus*) (Welker et al., 2009). In many Canidae including the dog, *V. zerda*, *V. vulpes*, *C. latrans* and in Pinnipedia, the cerebellum is nearly invisible from the dorsal aspect. Largely exposed cerebellum is observed in the cat (Smith et al., 2001). The cerebellar hemispheres division into lobuli was conducted only on the basis of macroscopic analysis (Figures 1A,B,D,E, 9D, 10B,D, 11B,D, 12B). LACR, LACC, LPm, PfD, PfV and Fc with PFc were clearly distinct. The lobules of the vermis were depicted also in MRI as part within the ventriculus and on the surface (Figures 1A,B,D,E, 9–12). Vm evinces relatively distinct division into its lobuli – all of them were depicted but the border between Dc and FVm is unclear. The noteworthy sections are in Figure 9D where PCM is continued into the pons and in Figure 10D wherein PCC is continued in MO. The folia cerebelli are apparent in both methods. In MRI they form alternating hypo- and hyperintense strips (Figures 9C, 10A,C, 11A,C, 12A). The hyperintensity are typical for cortex and nuclei of the cerebellum. In Figures 11A,D the cortex cerebelli due to indentation from the caudal aspect is also visible within the white matter so that it resembles nuclei.

#### Internal Anatomy

NF was depicted only in MAC (Figure 11B) as a round zone below the vermis. The nucleus fastigii is located medially and more prominently separated from NIC than NIC from NLC. In both methods, the NIC and the NLC were discernible. The borders between them are very subtle in MAC (Figures 10D, 11B) and sometimes indistinguishable in MRI, so that NLC and NIC form a common hyperintense mass and are collectively labeled in Figure 10C. However, in more caudal sections the distinction between NLC and NIC was more prominent (Figure 11A). The isolated NIC is also discernible in its rostral section in Figure 10A, however, the signal intensity is much lower. The relative volume and topography of the nuclei is comparable in the dog, after staining (Fletcher, 2007). All three nuclei were identified in MRI in the cat (Gray-Edwards et al., 2014), but only NLC in MRI in *Z. californianus* (Montie et al., 2009) and none of them, in MR,I in the dog (Leigh et al., 2008). The staining makes the structures easily discernible (Welker et al., 2009) and all the nuclei are present in Carnivora species available in CMBC.

### Medulla Oblongata

#### External Anatomy

In *U. arctos*, MO is dorso-ventrally flattened as in the dog (Whalen, 2003). Also Mettler and Goss (1946) found it in *A. melanoleuca* in similar shape. In comparison, MO of *Z. californianus* is nearly round in its cross-section in its caudal part (Montie et al., 2009). Cat MO seems to evince an intermediate state between those two extremes (Welker et al., 2009).

#### Gray Matter

The most prominent nuclei were NOv highly contrasted in MAC (Figures 10D, 11B,D) assuming a signature shape. Excellent contrast is apparent in MRI as a hyperintense, comma-shaped symmetrical zone with sharp borders (Figures 10C, 11A,C). In MAC and one MRI cross-section (Figure 11A) HNOv was discernible as a subtle white or hypointense string. HNOv are not bulged into olivae on the surface in *U. arctos.* HNOv is typical for a few mammalian orders including Carnivora (Welento, 2002) and was also visualized in MRI in the cat (Gray-Edwards et al., 2014) but not found in the dog (Leigh et al., 2008) or in *Z. californianus* (Montie et al., 2009). The shape of NOv in the cross-section varies among Carnivora species available in CMBC. In *U. arctos* and *U. maritimus* its medial part is wider and shorter whereas the lateral part is thinner and longer. In the dog, both parts are nearly equal in length but the medial one is wider. In *Z. californianus* the medial part is scant and the lateral part is considerably broader. NCL is another quite well defined nucleus taking up more dorsal residence above an apparent TSNT. It is also visible in MAC over relative large distance where it forms an exceptionally conspicuous zone of gray matter (Figures 10D, 11B) in Figure 11B. It is relatively less apparent in MRI, however, an initial TSNT identification facilitates the pinpointing of NCL. Similarly, NCM is conspicuous in MAC (Figures 11D, 12B) whereas in MRI its borders are unclear. The topography of NCM is consistent with that in Fletcher (2007) but due to large concentration of nuclei in the dorsal region of the medulla oblongata, we cannot rule out confusion with other nuclei, including nucleus parasympathicus nervi vagi, nucleus motorius nervi hypoglossi or nucleus tractus solitarii. On the other hand, NG is more distinguishable, in MRI, due to hyperintensity (Figure 11C) and as it forms TNG, identification in MAC is also effortless (Figure 11D). NVb were collectively depicted only in MAC as a distinct dark periventricular zone delineated by white matter of pedunculi cerebellares and NVII (Figure 10B). They are at the level of CT and consistent with topography described in literature (Kobryń and Kobryńczuk, 2004) and pointed by Fletcher (2007) in the dog. There is a unilateral highly hyperintense zone measuring about 4 mm in one MRI section (Figure 9A). It was unnoticeable in MAC, and due to its unilateral placement, we suppose it may be a lesion. NVb were depicted in the cat (Gray-Edwards et al., 2014) and in the dog (Leigh et al., 2008) and none of other nuclei of MO were depicted in abovementioned papers except for NOv.

#### White Matter

In the initial section of the medulla oblongata CT, we found a laterally extended band of white matter across MO width (Figure 10B). It is hypointense and devoid of ventral border in MRI, so the signal intermingles with that of TPy lying below (Figure 10A). The border of CT is also unclear in MAC. There are nerve fibers coursing from dorsal to ventral surface possible to spot in MAC but not in MRI: of NVII (Figure 10B), NXII (Figures 11B,D) and NVIII (Figure 10B). However, the latter was depicted in MRI in its free part on the ventro-lateral surface of MO (Figure 10A). The decussations of main neural tracts are discernable in both methods. LMs (Figures 10C,D, 11) are apparent centrally as a modest sphenoidal zone. LMs in MRI are hypointense with unclear borders; however, DLM occupies almost the entire central area of MO (Figure 11C). In MAC, TPy and DLM are found with well-defined boundaries (Figures 11B,D). DLM is reverse-Y-shaped, but for DPy the Y-shape is not reversed (Figure 12). The closing section of MO contains typical funiculi: FuD, FuV and FuL (Figure 12). In MRI they form a hypointense rim (Figure 12A). It is possible to notice a division of FuD into FaG and FaC in MAC (Figure 12B) whereas in MRI the division is not apparent. TSNT with NTSNT (Figures 9C,D, 10–12) are consistent across the entire course and they were also depicted in MRI in the cat (Gray-Edwards et al., 2014). The white matter of MO is generally consistent in all Carnivora groups according to specimens in CMBC.

### Reticular Formation

The area occupied by the reticular formation is difficult to inequivocally identify in MAC (Figures 8B,D, 9B,D, 10B,D, 11B,D). The only intimation of its presence is slightly darker coloration of the area. In MRI, the signal intensity is high enough to notice the broad character of the area (Figures 9A,C, 10A,C, 11A,C), but we could not delineate the exact borders of the area. In two MRI sections (Figures 9C, 10A) the central area is featured by apparently higher signal intensity – we suppose it comes from NRh and the nucleus rhaphe magnus as depicted by Fletcher (2007) in stained cross-sections in the dog.

### Ventricular System

The ventricular system in general is well discernible in both methods. In MAC it forms obvious empty spaces, in some sections very tight – rostral parts of VL (Figures 4B,D, 5B,D, 6B) or VT (Figure 6B). A broader lumen was observed in the middle of VL (Figures 7B,D), initial section of VT (Figure 6B), AqC (Figures 7D, 8B,D, 9B) and VQ (Figures 9D, 10B,D, 11B,D). In MRI the same features are observed for abovementioned sections and the entire ventricular system is completely devoid of signal. The signal decrement is typical for areas occupied by larger vessels (Figures 4C, 5A,C, 6A). In Figures 10C, 11B, respectively, a hyperintense or black zone is present – PCVQ. The lumen of VL in *U. arctos* is tightened in the rostral part to an arcuate cleft (Figures 4; 5, 6A,B) and the similar state is observed in the dog (Leigh et al., 2008), the cat (Gray-Edwards et al., 2014) and *Z. californianus* (Montie et al., 2009). In the ferret, the arcuate cleft is more asymmetrical – the dorsal arm is shorter (Sawada et al., 2013). In Figures 6A,B the foramen interventriculare was captured. The lumen of VT in Carnivora is reduced due to a large ATh (Kobryń and Kobryńczuk, 2004) and in *U. arctos* that is also evident (Figures 6C,D, 7A,B). Figure 7D shows the aditus ad aquaeductus cerebri with CCa lying above it. The canalis centralis was not apparent in a thorough examination of the most caudal parts of MO.

## Conclusion

1. Most of the recognized brain structures were apparent in both MRI and macroscopic anatomy. The tractus olfactorius medialis, corpus subthalamicum, brachium colliculi rostralis, fasciculus longitudinalis medialis, nuclei vestibulares, velum medullare rostrale, pedunculus flocculi, nucleus fastigii, fasciculus gracilis et cuneatus (nine structures) were depicted only in macroscopic anatomy analysis. The glandula pinealis, lemniscus lateralis and nuclei rhaphe (three structures) were visualized only in MRI. The lack of the glandula pinealis in anatomical analysis was omitted in cross-section.
2. The brain surface of *U. arctos* is more convoluted than in the cat, the dog and Mustelidae but lesser than in Pinnipedia. The homologies of the middle and rostral parts are less controversial than in caudal part.
3. A characteristic trait for Ursidae and Pinnipedia is presence of three gyri frontales and a lozenge in rostral part of the hemispheres. There is a difference in interpretation of the course of the sulcus cruciatus in *U. arctos* caudal or rostral to the gyrus frontalis superior. This is an important issue because higher development of rostral area to the sulcus cruciatus is assumed to take part in social behavior in *C. crocuta* and *P. leo*.
4. The rhinencephalon in *U. arctos* is organized in a manner typical for macrosmatic mammals. The pedunculi olfactori are stout, the tuberculum olfactorium is round and three tractus olfactorius are present including the tractus olfactorius intermedius. The lobus piriformis is relatively shorter and wider than in most Carnivora.
5. The claustrum in *U. arctos* brain cross-sections assumes a crescent-shape in contrast to the dog and cat where it assumes more pickaxe-shape. In MRI the claustrum usually resembles cerebral cortex thickening. The nucleus accumbens is more apparent in MRI. The boundaries between the globus pallidus and putamen are unclear in MRI and their signals usually mingle. The nucleus endopeduncularis is present and clearly evident in both methods.
6. The septum pellucidum in *U. arctos* is absent. Interestingly, it is present in most Carnivora including *U. maritimus*.
7. The hippocampal formation in *U. arctos* is divided into typical dorsal and ventral parts. The gray matter is largely reduced in dorsal part, however to a lesser extent than in Pinnipedia.
8. The epiphysis in *U. arctos* is relatively larger than in the dog but smaller than in Pinnipedia. It was visualized only in MRI. The hypophysis was not preserved. The corpora mammillaria are paired.
9. The layers of the colliculus rostralis are more evident in MRI in our study. The substantia grisea centralis is more contrasted in unstained cross-sections than in MRI where its signal intensity is weak.
10. Neither of methods used allowed to identify the nuclei of the pars dorsalis pontis in *U. arctos*. Out of the pars ventralis pontis the nuclei pontis were apparent.
11. The hemisphaeria cerebelli in *U. arctos* are prominent. The lobuli of the vermis and most lobuli of the hemispheres were depicted.
12. The boundary between the nucleus interpositus cerebelli and the nucleus lateralis in *U. arctos* is unclear. The nucleus fastigii was identified only in macroscopic anatomy method.
13. The medulla oblongata in *U. arcots* is largely dorso-ventrally flattened unlike the round in cross-section in Pinnipedia and slightly ovate in other Carnivora. Most of nuclei were impossible to discern in either method. The shape of nucleus olivaris in cross-section shows interspecies differences among Carnivora. The canalis centralis were undiscernible.

## Data Availability

MRI data in DICOM format are available in an online repository (https://gin.g-node.org/LukaszPasko/Ursus_arctos_RH0178_15_brain_MRI).

## Author Contributions

TS, ŁP, AS, and RM conceived the study. SR and DH collected the samples, then submitted by AS, RM, and ŁP to the Center of Experimental Diagnostics and Innovative Biomedical Technology, where MW and PP performed MRI. TS and ŁP performed the manual cross-sections and subsequent analysis of those and MRI images. TS led the figure preparations and wrote the manuscript, further completed and edited by ŁP, AS, and DH. All authors edited and approved the manuscript.

## Conflict of Interest Statement

The authors declare that the research was conducted in the absence of any commercial or financial relationships that could be construed as a potential conflict of interest.

## Supplementary Material

FIGURE S1 Phylogenetic relationship among Carnivora families with position of Ursidae marked by ^∗^ (after Flynn et al., 2005).

FIGURE S2 Gross brain anatomy of the brown bear (*Ursus arctos*), RH194/14 specimen. **(A)** dorsal surface; **(B)** ventral surface; **(C)** right lateral surface; **(D)** left lateral surface; **(E)** rostral surface; **(F)** caudal surface.

FIGURE S3 Gross brain anatomy of the brown bear (*Ursus arctos*), RH155/14 specimen. **(A)** dorsal surface; **(B)** ventral surface; **(C)** right lateral surface; **(D)** left lateral surface; **(E)** rostral surface; **(F)** caudal surface.

FIGURE S4 Gross brain anatomy of the brown bear (*Ursus arctos*), RH0156/13 specimen. **(A)** dorsal surface; **(B)** ventral surface; **(C)** right lateral surface; **(D)** left lateral surface.
