# Supplementary figures and images for: The Brain Anatomy of the Brown Bear (Carnivora, Ursus arctos L., 1758) Compared to That of Other Carnivorans: A Cross-Sectional Study Using MRI
Source: Front Neuroanat. 2019 Aug 29;13:79. doi: 10.3389/fnana.2019.00079 (PMC6727829; doi:10.3389/fnana.2019.00079)

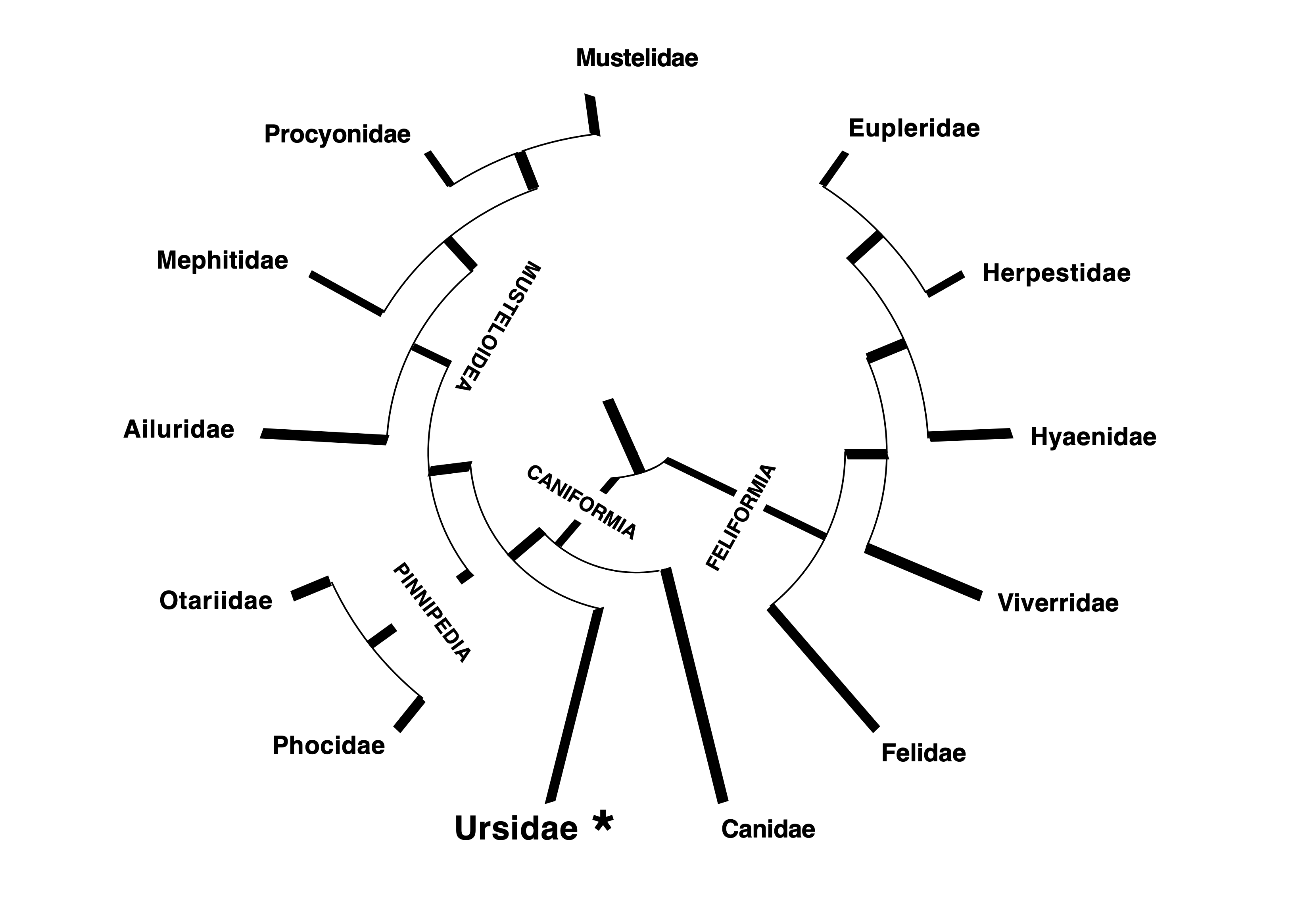

Supplement: FIGURE S1 — Phylogenetic relationship among Carnivora families with position of Ursidae marked by ∗ (after Flynn et al., 2005). [file Image_1.TIF]

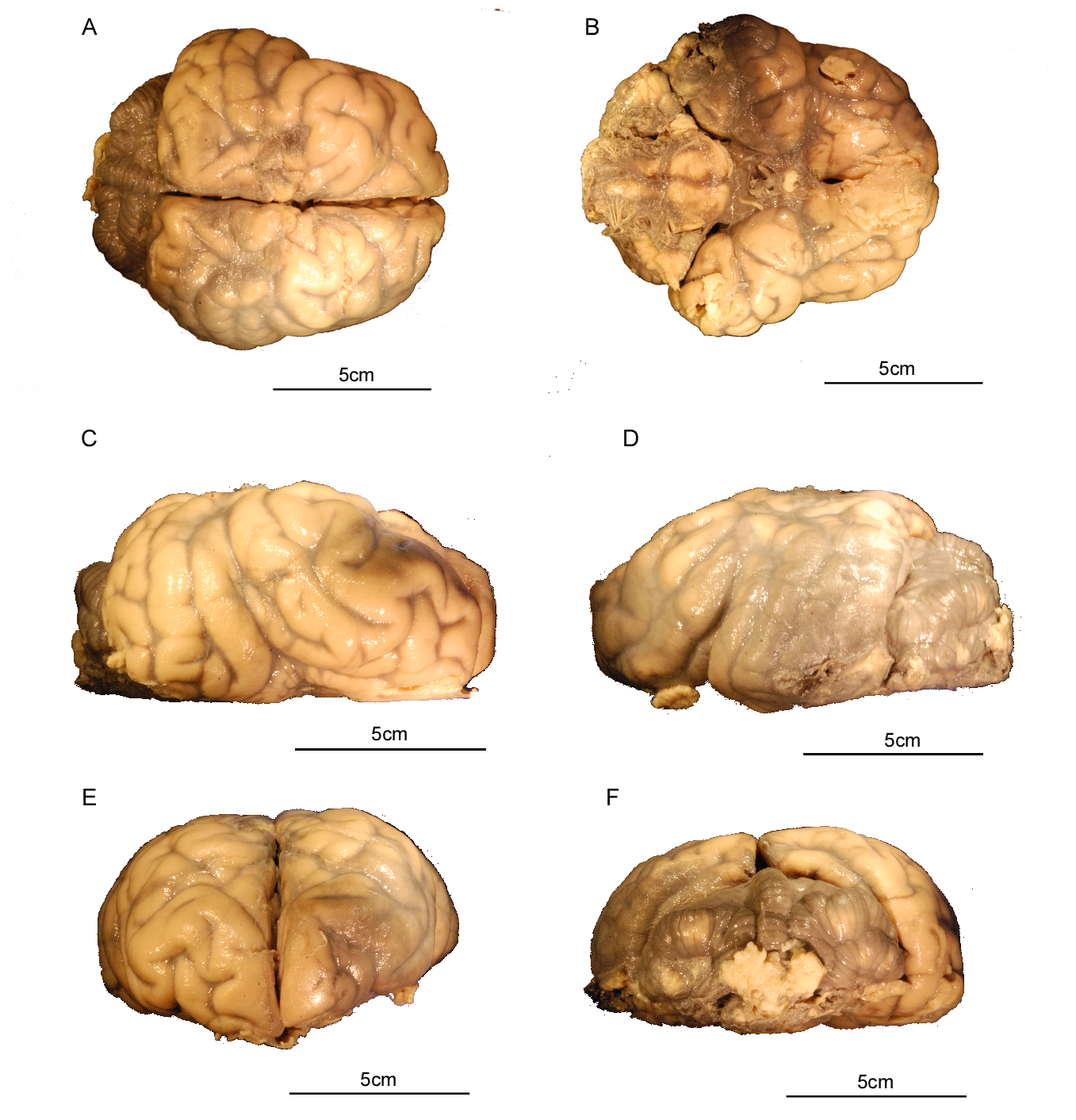

Supplement: FIGURE S2 — Gross brain anatomy of the brown bear (Ursus arctos), RH194/14 specimen. (A) dorsal surface; (B) ventral surface; (C) right lateral surface; (D) left lateral surface; (E) rostral surface; (F) caudal surface. [file Image_2.TIF]

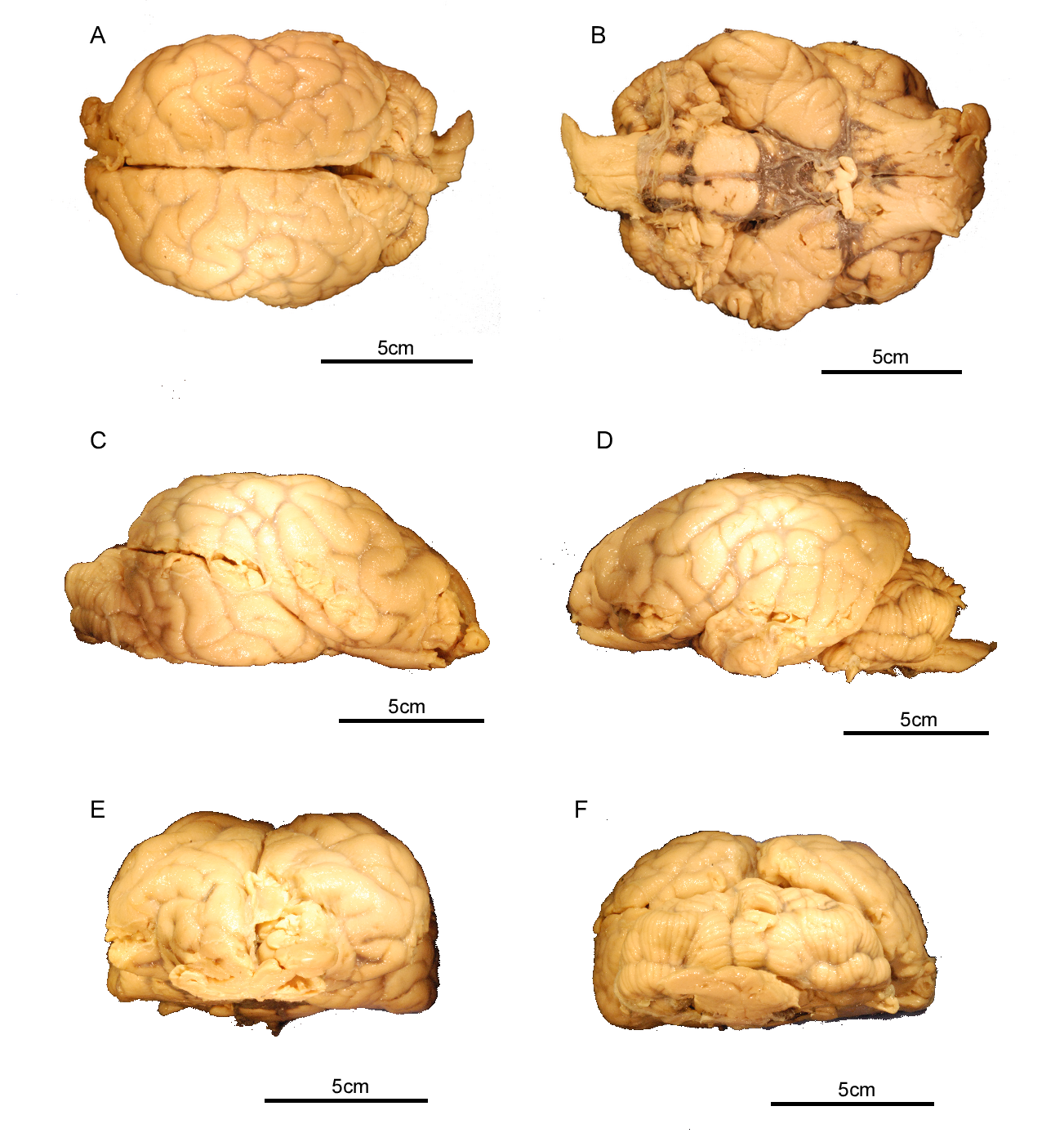

Supplement: FIGURE S3 — Gross brain anatomy of the brown bear (Ursus arctos), RH155/14 specimen. (A) dorsal surface; (B) ventral surface; (C) right lateral surface; (D) left lateral surface; (E) rostral surface; (F) caudal surface. [file Image_3.TIF]

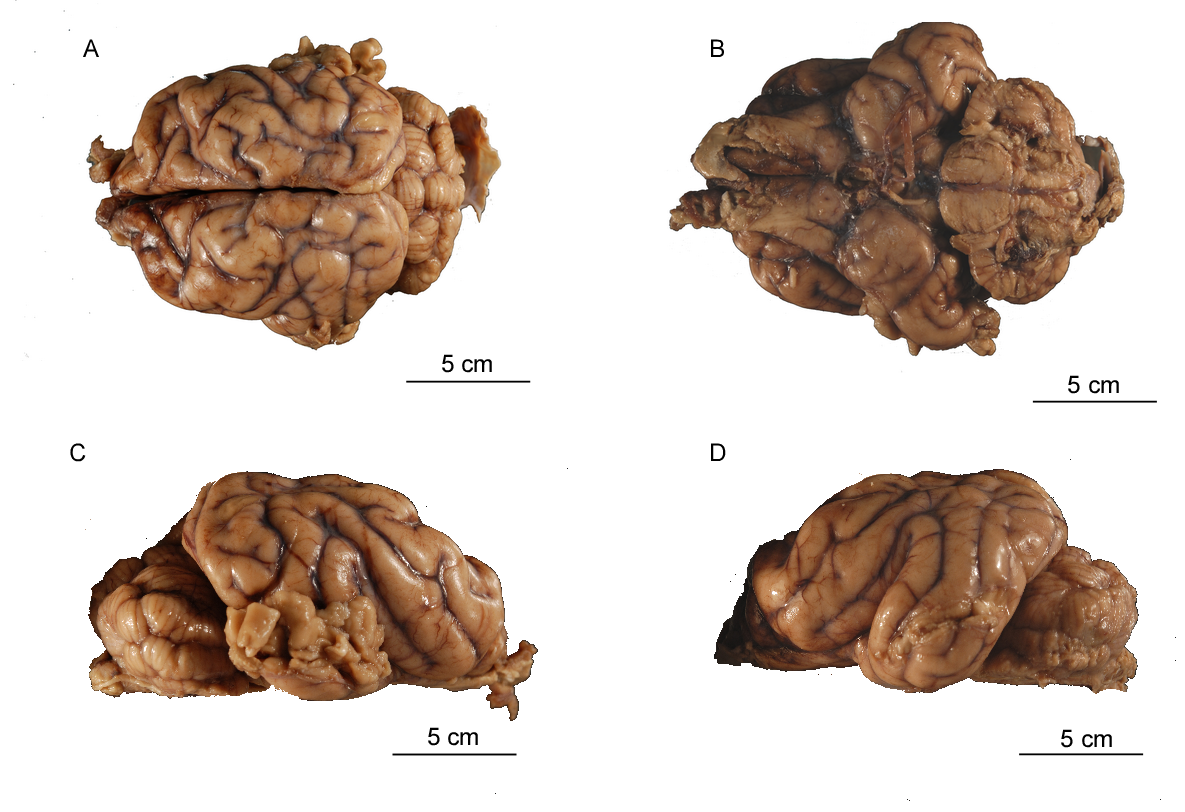

Supplement: FIGURE S4 — Gross brain anatomy of the brown bear (Ursus arctos), RH0156/13 specimen. (A) dorsal surface; (B) ventral surface; (C) right lateral surface; (D) left lateral surface. [file Image_4.TIF]
